# A SERPINA1-associated SOX4–FN1 epithelial program linked to lymphatic endothelial responses in aggressive papillary thyroid carcinoma

**DOI:** 10.3389/fcell.2026.1929991

**Published:** 2026-10-27

**Authors:** Yifan Zhong, Xiaorong Zhang, Qing Yang

**Affiliations:** 1 Department of Pathogenobiology, College of Basic Medical Sciences, Jilin University, Changchun, Jilin Province, China; 2 Department of Respiratory Medicine, Center of Pathogen Biology and Infectious Diseases, Key Laboratory of Organ Regeneration and Transplantation of The Ministry of Education, State Key Laboratory for Diagnosis and Treatment of Severe Zoonotic Infectious Diseases, The First Hospital of Jilin University, Changchun, China

**Keywords:** lymphatic endothelial cells, multi-omics integration, papillary thyroid carcinoma, SERPINA1, tumor microenvironment

## Abstract

**Background:**

Lymph node metastasis (LNM) complicates risk assessment and treatment planning in papillary thyroid carcinoma (PTC). How tumor epithelial programs relate to the lymphatic microenvironment remains unclear.

**Methods:**

We integrated single-cell RNA sequencing and spatial transcriptomic data with bulk cohorts to characterize epithelial states and their clinical associations. Bulk cohorts came from The Cancer Genome Atlas (TCGA) and Gene Expression Omnibus (GEO). Deconvolution, pathway, regulatory, and cell–cell communication analyses examined epithelial–lymphatic relationships; machine learning was used to construct an LNM classifier. We tested key findings using perturbation and rescue experiments, lymphatic endothelial cell (LEC) assays, xenografts, and human tissue analyses.

**Results:**

An atlas of 221,187 cells from 46 thyroid specimens identified an extracellular matrix-associated THY5 epithelial state. THY5 was enriched in pooled cells from aggressive tumors, although its sample-level abundance did not differ significantly between tumor groups. Bulk analyses linked higher estimated THY5 abundance to adverse clinicopathological features and immune exclusion. An exploratory 11-gene classifier incorporating SERPINA1 and FN1 discriminated lymph node status. Areas under the receiver operating characteristic curve were 0.810 in the TCGA internal evaluation set and 0.768 and 0.800 in two GEO cohorts. Communication and spatial analyses implicated FN1-related epithelial–lymphatic interactions and local THY5–LEC co-occurrence. Perturbation experiments supported functional relationships among SERPINA1, SOX4, and FN1. SOX4 re-expression increased FN1 mRNA and protein levels relative to SERPINA1 knockdown alone. FN1 re-expression in SERPINA1-depleted tumor cells counteracted reductions in LEC markers, tube formation, and migration. SERPINA1 knockdown also suppressed xenograft growth and reduced FN1 staining.

**Conclusion:**

Our findings support functional links among SERPINA1-associated SOX4–FN1 expression, malignant epithelial behavior and LEC responses in PTC, highlighting the potential of this molecular program for investigating epithelial–lymphatic interactions. The 11-gene LNM classifier discriminated lymph node status across the evaluated cohorts, providing an exploratory framework for molecularly informed risk assessment.

## 1 Introduction

Papillary thyroid carcinoma (PTC) is the most common endocrine malignancy and usually has a favorable prognosis (Cancer Genome Atlas Research Network, 2014; Fagin and Wells, 2016; Lim et al., 2017; Tiedje and Fagin, 2020; Li C. et al., 2025). Nevertheless, some tumors invade locally, spread to regional lymph nodes, or recur (Liu et al., 2017; Guo et al., 2025). Cervical lymph node metastasis (LNM) is common at diagnosis and informs surgical and surveillance decisions. Its association with locoregional recurrence depends in part on nodal number, size, and extranodal extension (Randolph et al., 2012; Ringel et al., 2025; Xiao et al., 2025). Routine assessment can miss occult nodal disease and micrometastases (Arturi et al., 1997; Qubain et al., 2002). Ultrasound findings can also be inconclusive in small or anatomically constrained nodes (Leboulleux et al., 2007; Ringel et al., 2025).

PTC progression involves both epithelial heterogeneity and microenvironmental remodeling. Single-cell and spatial studies have resolved diverse epithelial states, immune compartments, fibroblast programs, and endothelial populations in thyroid cancer (Pan et al., 2022; Ji et al., 2025; Liao et al., 2025; Zhan et al., 2025). Compared with immune and stromal remodeling, the relationship between epithelial states and lymphatic remodeling is less well characterized. This gap persists despite the established role of lymphangiogenesis and lymphatic vessel remodeling in metastatic dissemination (Stacker et al., 2014; de Visser and Joyce, 2023; Pan et al., 2024). How malignant epithelial states relate to lymphatic endothelial cells (LECs) in LNM-prone tumors therefore remains an open question.

Bulk transcriptomic cohorts can link gene programs to lymph node status but cannot identify their cellular origin. Single-cell RNA sequencing (scRNA-seq) resolves epithelial states but loses tissue architecture, whereas spatial transcriptomics (ST) localizes expression programs with limited single-cell resolution (Ståhl et al., 2016; Rao et al., 2021; Cable et al., 2022). Combining these data with experimental testing can help examine epithelial–lymphatic relationships. Extracellular matrix (ECM) programs involving fibronectin 1 (FN1) and integrins have been linked to PTC biology and clinical features (Liu et al., 2025; Rocco et al., 2025). Whether an FN1-centered epithelial program connects an LNM-prone malignant state to LEC activation remains unresolved.

We integrated scRNA-seq and ST data with bulk cohorts from The Cancer Genome Atlas (TCGA) and Gene Expression Omnibus (GEO). Bayesian deconvolution and LNM-oriented gene prioritization were used to examine the clinical associations of epithelial states and identify an FN1-associated program. Perturbation and rescue experiments tested functional relationships among serpin family A member 1 (SERPINA1), SRY-box transcription factor 4 (SOX4), and FN1. LEC assays, spatial analyses, and tissue analyses examined how this tumor-cell program related to LEC responses, including nuclear factor κB (NF-κB)-related protein changes.

## 2 Materials and methods

### 2.1 Data sources, study design and phenotype definitions

Public scRNA-seq, ST, and bulk transcriptomic datasets were integrated for PTC analysis. The scRNA-seq datasets comprised GSE184362, GSE193581, GSE250521 and GSE281736. Spatial analyses used Visium sections from GSE250521. Bulk analyses included the TCGA thyroid carcinoma (TCGA-THCA) cohort and 12 GEO thyroid transcriptomic cohorts: GSE83560, GSE112202, GSE126698, GSE138042, GSE153659, GSE165724, GSE193623, GSE201365, GSE202413, GSE213647, GSE223765 and GSE237841, comprising 859 samples. GSE129562 was additionally used for LNM modeling. Aggressive PTC was defined from the available metadata as pathological T stage > T3b, locally invasive PTC or the diffuse sclerosing variant.

### 2.2 scRNA-seq processing and annotation

Single-cell data were processed using Seurat. Potential doublets and low-quality cells or genes were removed before normalization. Harmony was used for data integration where applicable, followed by clustering and uniform manifold approximation and projection (UMAP) visualization. Cell types were annotated using SingleR-assisted labels, canonical marker genes and manual curation.

### 2.3 Epithelial compartment and cell-state analyses

Epithelial cells were extracted and reprocessed for subclustering, marker identification, cell-composition analysis and cell-state scoring. Augur and scDist were used to assess transcriptional differences associated with clinical phenotypes. Copy-number-associated expression patterns and genomic instability were assessed using inferCNV and genomicInstability, respectively. CytoTRACE2 was used to estimate developmental potential, and ROGUE was used to assess transcriptional homogeneity within cell populations. For sample-level THY5 analysis, abundance was defined as the number of THY5 cells divided by the total number of epithelial cells in each tissue sample. Samples without THY5 cells were assigned zero abundance and retained in the analysis. THY5 detection was defined as the presence of at least one annotated THY5 cell in a tissue sample. Aggressive and non-aggressive tumors were compared using a two-sided Wilcoxon rank-sum test, with tissue samples as the units of analysis. To assess unequal sample contributions, samples within each group were ranked by THY5 cell count. The fraction of all THY5 cells contributed by the three largest contributors in each group was then calculated.

### 2.4 Differential expression, pathway enrichment and trajectory analysis

Differential expression in single-cell and spatial data was analyzed using Seurat/SCOP workflows, and bulk differential expression was analyzed using DESeq2. Over-representation analysis and gene set enrichment analysis (GSEA) were performed with clusterProfiler and Molecular Signatures Database (MSigDB) gene sets, accessed through msigdbr where applicable. Gene-set activity was evaluated using irGSEA-associated methods. Partition-based graph abstraction (PAGA) was used to assess connectivity among cell populations, and Monocle3 was used for trajectory reconstruction and pseudotime ordering.

### 2.5 Bulk deconvolution, clinical associations and predictive modeling

Bulk transcriptomic cohorts were deconvolved with BayesPrism using the single-cell dataset as the reference. Associations between estimated cell-population abundance and clinicopathological variables were examined. Immune-response scores and predicted drug responses were evaluated using Tumor Immune Dysfunction and Exclusion (TIDE) and oncoPredict, respectively. For abundance-based comparisons, tumor samples were divided into high- and low-abundance groups using the median estimated abundance within each cohort.

Candidate genes for LNM prediction were selected by integrating weighted gene co-expression network analysis (WGCNA) with differential expression. In the GEO and TCGA cohorts, genes were selected from WGCNA modules whose eigengenes were positively correlated with both PTC status and the estimated abundance of the epithelial subpopulation of interest. Within these modules, genes associated with PTC status at a gene-level nominal P < 0.05 were retained, and genes shared between the two cohorts were identified. These shared genes were then intersected with genes upregulated across four comparisons: tumor versus normal tissue in TCGA and GEO, and high versus low estimated epithelial-subpopulation abundance in TCGA and GSE138042. Candidate genes represented in the TCGA, GSE138042 and GSE129562 expression matrices were retained for modeling.

TCGA samples with defined N0 or N1 status were divided into training and internal evaluation sets by stratified sampling at a 70:30 ratio. GSE138042 and GSE129562 were used for external evaluation. Gene expression was standardized to z-scores separately within the training set, internal evaluation set and each external cohort. Using the candidate genes, different feature-selection methods were combined with machine-learning classification algorithms to construct models for predicting lymph node status in the training set. Predictive performance was compared in the internal evaluation set and external cohorts. Receiver operating characteristic (ROC) curves and the area under the curve (AUC) were used to assess discrimination. Calibration curves and decision curve analysis (DCA) were generated from the selected model predictions to assess calibration and potential clinical net benefit. Candidate screening preceded the TCGA split, and evaluation-cohort AUCs were used when comparing data splits and model combinations.

### 2.6 Ligand–target and cell–cell communication analyses

NicheNet was used for receiver-centered ligand–target prioritization (Browaeys et al., 2020). Candidate ligands were ranked using ligand–target regulatory potential and expression information from the specified sender and receiver populations. CellChat was used to infer ligand–receptor communication from normalized expression matrices and cell-type annotations (Jin et al., 2021). Communication networks were analyzed overall and separately by clinical group, including interaction counts, communication strengths and signaling roles of sender and receiver populations.

### 2.7 Spatial transcriptomics, deconvolution and niche analyses

Spatial transcriptomic sections were processed as Seurat spatial objects, including spot-level quality control, normalization, clustering, marker identification and enrichment analysis. Cell-type proportions were estimated using robust cell type decomposition (RCTD), implemented in spacexr, with the single-cell dataset as the reference (Cable et al., 2022). SpatialCellChat was used to assess ECM–receptor communication, with pathways ranked by information flow and spatial hotspots identified using Getis–Ord Gi analysis. Composition clustering and cell-type co-occurrence were analyzed with ISCHIA, neighborhood contributions were evaluated with MISTy, and spatially variable programs were assessed with SpaGene.

### 2.8 Regulon inference and *in silico* perturbation

Single-cell regulatory network inference and clustering (SCENIC) was used to infer transcription-factor regulons using GENIE3, RcisTarget and AUCell (Aibar et al., 2017). Regulon–target annotations, regulon activity and expression correlations were used to evaluate candidate regulatory relationships. SOX4 virtual knockout used scTenifoldKnk v1.0.3 with scTenifoldNet v1.3, whereas virtual overexpression used a custom scTenifoldOE function. Input comprised raw counts from aggressive-tumor THY5 cells for 1,500 highly variable genes selected in the epithelial dataset. Both analyses used 10 networks, 500 cells sampled with replacement per network, and three principal components. Knockout removed outgoing SOX4 edges with random seed 7, whereas overexpression increased their weights tenfold. Differentially regulated genes were identified at a false discovery rate (FDR) < 0.05.

### 2.9 Cell culture, transfection and co-culture

BCPAP and TPC-1 cells were cultured in RPMI-1640 medium supplemented with 10% fetal bovine serum (FBS). LECs were maintained in endothelial cell-specific medium supplemented with serum. All cells were cultured under sterile conditions in a humidified incubator at 37 °C with 5% CO_2_.

For gene knockdown, overexpression and rescue experiments, cells were subjected to transient small interfering RNA (siRNA)-mediated knockdown, plasmid-mediated overexpression or stable knockdown, as indicated in the corresponding experiments. Cells were collected for molecular detection, imaging or functional assays at the indicated endpoints. The sequences of the siRNAs and single-guide RNAs (sgRNAs), as well as the plasmid maps used in this study, are provided in Supplementary Material 1.

For tumor cell–LEC co-culture experiments, tumor cells were seeded in the upper chambers of Transwell inserts and LECs in the lower chambers for LEC tube-formation and cell migration assays. For LEC Transwell assays, LECs were seeded in the upper chambers and tumor cells in the lower chambers. Alternatively, LECs were treated with conditioned medium (CM) collected from the corresponding tumor cell groups. Co-culture and CM experiments were subsequently used for protein expression analysis and related cell-based assays.

### 2.10 Western blotting, co-immunoprecipitation and immunoprecipitation–mass spectrometry

Total cellular proteins were extracted from treated tumor cells or LECs using radioimmunoprecipitation assay (RIPA) lysis buffer supplemented with protease and phosphatase inhibitors. Cell lysates were clarified by centrifugation, and protein concentrations were determined using a protein assay. Proteins were mixed with sample loading buffer, denatured and separated by sodium dodecyl sulfate–polyacrylamide gel electrophoresis (SDS-PAGE). They were then transferred onto polyvinylidene difluoride (PVDF) membranes. Membranes were blocked and incubated with the indicated primary antibodies, followed by appropriate horseradish peroxidase (HRP)-conjugated secondary antibodies. Protein signals were visualized using an enhanced chemiluminescence (ECL) detection system. Band intensities were quantified using ImageJ and normalized to the corresponding loading control. For SOX4 re-expression in SERPINA1-depleted cells (Supplementary Figures S10C,D), FN1, SERPINA1 and SOX4 band intensities were normalized to glyceraldehyde-3-phosphate dehydrogenase (GAPDH). The resulting relative protein values were plotted without further normalization to the control-group mean.

The primary antibodies used in this study are described below, including their suppliers, catalogue numbers, clones and reference western blot dilutions, where available. The SERPINA1/alpha-1-antitrypsin antibody was rabbit polyclonal (Proteintech, 16382-1-AP; reference western blot dilution, 1:30000). The FN1/fibronectin antibody was mouse monoclonal immunoglobulin G1 (IgG1), clone 1G10F9 (Proteintech, 66042-1-Ig; reference western blot dilution, 1:6000). The O-linked N-acetylglucosamine transferase (OGT) antibody was mouse monoclonal, clone 2B2A6 (Proteintech, 66823-1-Ig; reference western blot dilution, 1:10000). The O-linked N-acetylglucosamine (O-GlcNAc) antibody was mouse monoclonal RL2 (Abcam, ab2739), suitable for western blotting and immunocytochemistry/immunofluorescence. The SOX4 antibody was rabbit polyclonal (Proteintech, 27414-1-AP; reference western blot dilution, 1:1500). The PROX1 antibody was rabbit polyclonal (Proteintech, 11067-2-AP; reference western blot dilution, 1:5000). The FLT4/vascular endothelial growth factor receptor 3 (VEGFR3) antibody was rabbit polyclonal (Proteintech, 20712-1-AP; reference western blot dilution, 1:300). The NF-κB p65 antibody was mouse monoclonal, clone 1B12D11 (Proteintech, 66535-1-Ig; reference western blot dilution, 1:1000). The phospho-NF-κB p65 (Ser468) antibody was rabbit recombinant monoclonal, clone 6N1 (Proteintech, 82335-1-RR; reference western blot dilution, 1:5000).

Co-immunoprecipitation (co-IP) was performed under non-denaturing lysis conditions. Equal amounts of cell lysate were pre-cleared and incubated with target antibodies or matched immunoglobulin G (IgG) controls, followed by capture with Protein A/G beads. Immunoprecipitated material and input lysates were analyzed by western blotting using reciprocal antibodies.

For immunoprecipitation–mass spectrometry (IP-MS), immunoprecipitated protein complexes were prepared as described above and analyzed by a mass-spectrometry service provider. The resulting protein identification and summary tables were used to nominate proteins associated with the immunoprecipitated bait for subsequent validation and functional studies. The complete protein identification and summary data are provided in Supplementary Table S1.

### 2.11 Immunofluorescence and proximity ligation assay

For immunofluorescence (IF) analysis, cells were seeded on coverslips or confocal dishes and fixed with 4% paraformaldehyde. Cells were subsequently permeabilized and blocked before incubation with the indicated primary antibodies. After washing, cells were incubated with species-appropriate fluorescently labeled secondary antibodies. Nuclei were counterstained with 4′,6-diamidino-2-phenylindole (DAPI). Fluorescence images were acquired using a confocal laser-scanning microscope under identical acquisition settings within each experimental group. Representative images were processed and analyzed using ImageJ, where applicable. Detailed imaging parameters are provided in the corresponding source data or Supplementary Material.

For the *in situ* proximity ligation assay (PLA), cells were processed using a commercially available cell-based PLA kit according to the manufacturer’s instructions. Briefly, cells were fixed, permeabilized, and blocked, followed by incubation with the indicated pair of primary antibodies. After washing, PLA probes were added, and proximity-dependent signal generation was performed through probe hybridization, ligation, and rolling-circle amplification according to the manufacturer’s protocol. Nuclei were counterstained with DAPI, and PLA signals were visualized using confocal microscopy. Quantification was performed using ImageJ and expressed as the number of PLA puncta per cell or PLA signal density per field, as indicated in the corresponding experiments.

### 2.12 RT-qPCR and ChIP-qPCR

For reverse transcription quantitative polymerase chain reaction (RT-qPCR), total RNA was extracted from treated cells. Purified RNA was reverse-transcribed into complementary DNA (cDNA), and quantitative PCR (qPCR) was performed using a SYBR Green-based detection system. The expression RT-qPCR assays measured SERPINA1, SOX4 and FN1 mRNA, with GAPDH as the reference gene. Relative gene expression levels were calculated using the comparative cycle threshold (Ct; 2^−ΔΔCT^) method. Expression RT-qPCR primer sequences and reference-gene information are provided in Supplementary Material 1, Section C.

Chromatin immunoprecipitation followed by qPCR (ChIP-qPCR) was performed to determine the occupancy of SOX4 at the regulatory region of the FN1 gene. Briefly, cells were cross-linked to preserve protein–DNA interactions and subsequently lysed. Chromatin was fragmented by sonication to generate DNA fragments of appropriate size. The sheared chromatin was then immunoprecipitated with an anti-SOX4 antibody or the corresponding matched IgG control. Following immunoprecipitation, immune complexes were recovered, cross-links were reversed, and the DNA was purified for subsequent qPCR analysis. The FN1-999 primer pair (targeting the FN1 promoter region containing a predicted SOX4-binding site) is provided in Supplementary Material 1, Section B.

### 2.13 Tumor-cell functional assays

Tumor cell proliferation and migration were evaluated using colony formation, 5-ethynyl-2′-deoxyuridine (EdU) incorporation, wound-healing, and Transwell assays.

For the colony-formation assay, treated tumor cells were seeded at a low density and cultured under the indicated conditions until visible colonies formed. Cells were then fixed and stained with crystal violet, and colonies were counted for the experiments in Supplementary Figures S10A,B.

For EdU incorporation assays, treated cells were incubated with EdU working solution for the indicated period. After fixation and permeabilization, incorporated EdU was fluorescently labeled using a click chemistry-based detection system. Nuclei were counterstained with DAPI, and the fraction of EdU-positive cells was quantified by fluorescence microscopy (Supplementary Figures S10A,B).

For wound-healing assays, cells were cultured to confluence to form a uniform monolayer. A linear scratch was generated using a sterile pipette tip, followed by gentle washing to remove detached cells. Cells were then maintained in serum-reduced or serum-free medium, as appropriate, during the migration period. Images were acquired at 0 and 24 h, and wound closure was quantified using ImageJ. Tumor-cell results are expressed as wound closure fractions (Supplementary Figures S10A,B). LEC wound-healing measurements were divided by the matched control value within each independent experiment and are reported relative to control (Supplementary Figure S10E).

For Transwell migration assays, treated cells were suspended in the appropriate medium and seeded into the upper chambers of Transwell inserts, while the lower chambers contained medium supplemented with the indicated chemoattractant. After incubation for the specified period, non-migrated cells were removed from the upper surface of the membrane. Cells that had migrated to the lower surface were fixed, stained, and counted in randomly selected microscopic fields.

### 2.14 LEC responses and tube-formation assays

LEC responses to tumor cell-derived CM or tumor-cell co-culture were assessed by western blotting for lymphatic endothelial and signaling markers and by functional migration assays. For tube formation, chilled Matrigel or an equivalent basement-membrane matrix was added to pre-cooled plates and allowed to polymerize. LECs were then seeded onto the polymerized matrix in the corresponding treatment medium. After incubation for the indicated period, tube-like structures were imaged using an inverted or fluorescence microscope, as appropriate. Tube-formation measurements were expressed relative to control, whereas Transwell migration was reported as the number of migrated LECs (Supplementary Figure S10E).

### 2.15 Xenograft assay, tissue microarray and immunohistochemistry

All animal experiments were reviewed and approved by the Experimental Animal Ethics Committee of the School of Basic Medical Sciences, Jilin University. Control tumor cells or tumor cells with stable gene knockdown were harvested during logarithmic growth and resuspended in sterile buffer or serum-free medium. The cells were then injected subcutaneously into BALB/c nude mice. At the experimental endpoint, mice were euthanized according to the approved protocol. Tumors were excised, photographed, weighed and measured along the maximum diameter, then collected for histological and immunohistochemical analyses.

Human thyroid tissue microarrays were obtained from Shanghai Outdo Biotech Co., Ltd. (Shanghai, China) with ethics approval from the provider. Tissue sections were deparaffinized and rehydrated, followed by antigen retrieval and blocking. Sections were then incubated with the indicated primary antibodies, followed by incubation with appropriate HRP-conjugated secondary antibodies. Immunoreactivity was visualized using 3,3′-diaminobenzidine (DAB) as the chromogenic substrate, and nuclei were counterstained with hematoxylin. Staining was evaluated by comparing tumor tissues with the corresponding adjacent thyroid tissues. Quantitative analysis of immunohistochemistry (IHC) staining was performed using mean optical density, positive staining area, or region-of-interest (ROI)-based intensity measurements, as appropriate for the corresponding experimental panel.

### 2.16 Statistical analysis

Statistical analyses were performed according to the study design and corresponding bioinformatic workflows. Experimental data are presented as mean ± standard error of the mean (SEM) or standard deviation (SD), as specified in the figure legends. Two-sided t-tests, analysis of variance (ANOVA), or nonparametric tests were used as appropriate. Multiple comparisons were adjusted using the Benjamini–Hochberg or Holm method where applicable. Sample sizes, specific tests, and correction procedures are provided in the relevant methods or figure legends. Unless otherwise stated, P < 0.05 was considered statistically significant.

## 3 Results

We combined multi-omics integration with experimental validation, incorporating scRNA-seq, ST, and TCGA and GEO transcriptomic cohorts, followed by *in vitro* functional assays and *in vivo* xenograft experiments. The data sources for the integrated analyses are shown in Figure 1A, and the overall study workflow is presented in Supplementary Figure S1.

**FIGURE 1:**
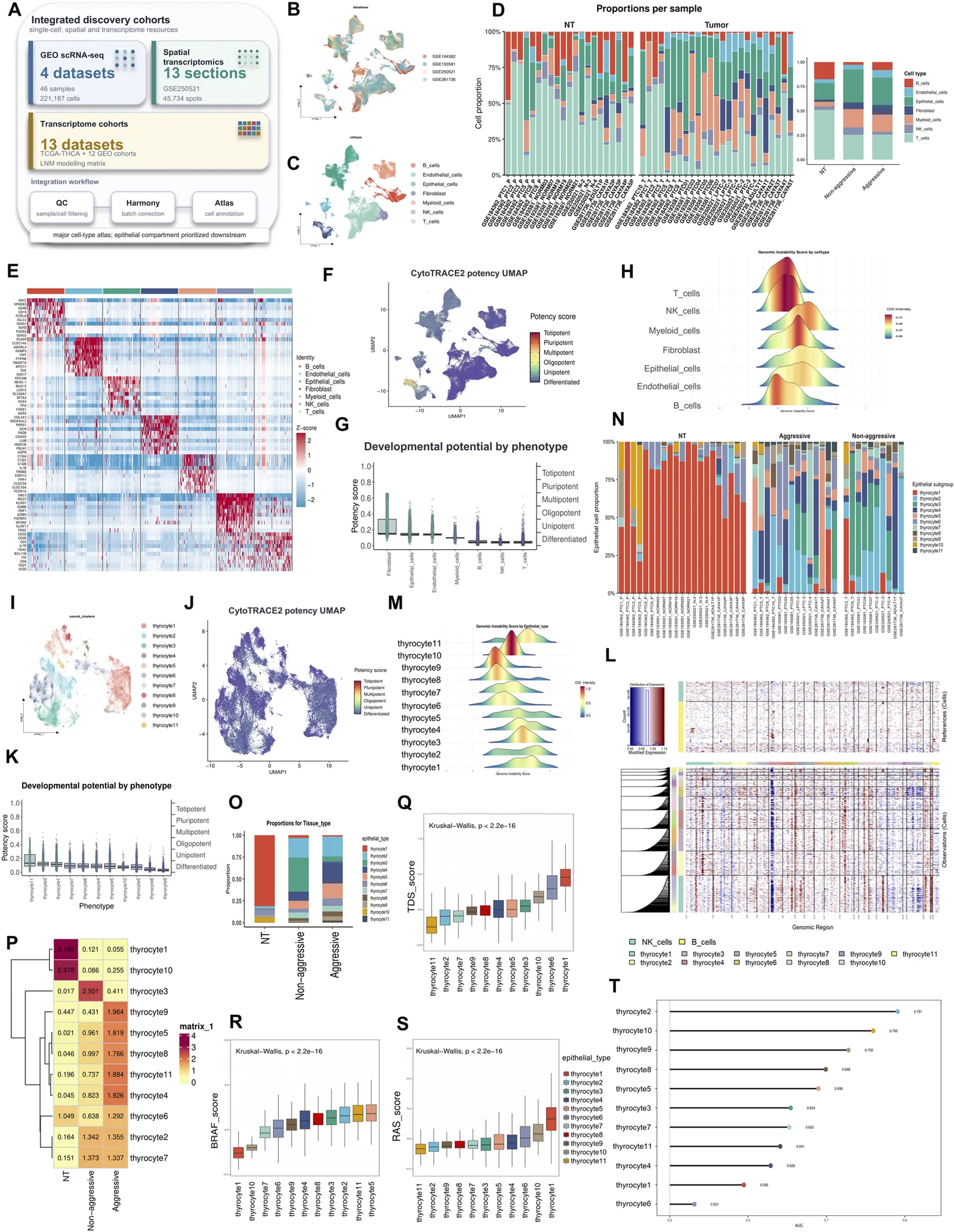
Single-cell atlas and characterization of epithelial subpopulations in PTC. **(A)** Public data sources and integration workflow. **(B)** Integrated UMAP colored by source dataset. **(C)** UMAP colored by major cell-type annotation. **(D)** Major cell-type proportions by sample and tissue group. **(E)** Cell-type marker expression heatmap. **(F, G)** CytoTRACE2 UMAP and score distributions across major cell populations. **(H)** Genomic instability score distributions across major cell populations. **(I)** UMAP of epithelial subpopulations THY1–THY11. **(J, K)** CytoTRACE2 UMAP and scores across epithelial subpopulations. **(L)** Inferred CNV heatmap with immune cells as the reference. **(M)** Genomic instability scores across epithelial subpopulations. **(N)** Epithelial subpopulation proportions in individual samples, grouped as NT, aggressive, and non-aggressive tissues. **(O)** Pooled epithelial subpopulation proportions by tissue group; these cell-count summaries are distinct from the sample-level THY5 comparison reported in the Results. **(P)** Ratios of observed to expected cell counts (Ro/e) by tissue group. **(Q–S)** Thyroid differentiation, BRAF-related, and RAS-related scores, respectively. **(T)** Augur analysis comparing aggressive and non-aggressive tumors across epithelial subpopulations. NT, normal thyroid; PTC, papillary thyroid carcinoma; UMAP, uniform manifold approximation and projection; CNV, copy-number variation.

### 3.1 Single-cell atlas and epithelial subpopulations in PTC

#### 3.1.1 Single-cell atlas and major cell populations

To characterize cellular heterogeneity and the thyroid cancer microenvironment, we integrated four public scRNA-seq datasets after stringent quality control and corrected between-sample batch effects using Harmony. The resulting atlas contained 221,187 cells from 46 thyroid specimens (Figures 1A,B; Supplementary Figures S2A,B). Unsupervised clustering and canonical marker expression identified seven major populations: epithelial cells, T cells, B cells, natural killer (NK) cells, myeloid cells, endothelial cells, and fibroblasts (Figure 1C). Annotation was supported by marker expression patterns (Figure 1E; Supplementary Figure S2C): EPCAM, KRT8, KRT18, and KRT19 in epithelial cells; CD3D, CD3E, CD2, and IL7R in T cells; CD79A, MS4A1, CD19, and IGHM in B cells; NKG7, GNLY, KLRD1, and NCAM1 in NK cells; LYZ, CD68, CD14, and FCGR3A in myeloid cells; PECAM1, VWF, CDH5, and CLDN5 in endothelial cells; and COL1A1, COL1A2, DCN, and LUM in fibroblasts.

Cellular composition varied with tissue status and disease aggressiveness (Figure 1D). At the individual-sample level, normal tissues generally contained a higher proportion of T cells, whereas several tumors showed increased relative proportions of epithelial cells, myeloid cells, and fibroblasts; some tumors also contained substantial endothelial fractions. When samples were summarized as normal thyroid (NT), non-aggressive, and aggressive groups, both tumor groups had higher epithelial and myeloid proportions and lower T-cell proportions than NT. Epithelial cells were most abundant in the non-aggressive group and less abundant in the aggressive group, although their proportion remained above that in NT. Myeloid proportions remained elevated in both tumor groups, whereas T-cell proportions were similar between the two tumor groups and lower than in NT. Endothelial and fibroblast proportions increased from NT through non-aggressive to aggressive tissues.

We next assessed developmental potential using CytoTRACE2 and examined genomic instability scores. Fibroblasts generally had the highest CytoTRACE2 scores, followed by epithelial and endothelial cells, whereas T cells, NK cells, and B cells had lower scores (Figures 1F,G). Genomic instability scores showed distinct distributions across cell types, with epithelial and endothelial cells both spanning broad ranges (Figure 1H).

#### 3.1.2 Epithelial subpopulations and THY5 characteristics

Reclustering epithelial cells identified 11 transcriptionally distinct subpopulations, designated THY1–THY11 (Figure 1I; Supplementary Figure S2D). CytoTRACE2 scores were relatively high in THY11, THY3, and THY1 and low in THY8 and THY9, with THY5 occupying an intermediate range (Figures 1J,K). Inferred copy-number variation (CNV), using immune cells as the reference, revealed distinct regional chromosomal expression shifts across epithelial subpopulations (Figure 1L). CNV scores differed significantly overall, with higher scores in THY6, THY9, and THY8 and moderately high scores in THY5 (Supplementary Figure S2E). Genomic instability score distributions also differed among epithelial subpopulations (Figure 1M). THY8 and THY9 had their main peaks at lower scores, whereas the peaks for THY1, THY2, and THY11 were shifted toward higher scores. THY10 showed a narrow, unimodal distribution. In contrast, THY5 displayed a broader distribution with several local peaks extending into higher score ranges.

Epithelial subpopulation composition varied among individual samples within the tissue groups (Figure 1N) and differed in the pooled tissue-group summaries. NT was dominated by THY1 and contained a higher THY10 proportion than either tumor group. THY3 was the largest epithelial component in non-aggressive tumors, followed by THY2. Compared with non-aggressive tumors, aggressive tumors had a lower THY3 proportion and higher proportions of THY4, THY5, THY8, THY9, and THY11, while THY2 proportions were similar (Figure 1O). In these pooled summaries, THY5 was scarce in NT and more abundant in aggressive than in non-aggressive tumors. To assess tissue preferences, we calculated the ratio of observed to expected cell counts (Ro/e; Figure 1P). THY1 and THY10 were enriched in NT, with Ro/e values of 3.189 and 2.978, respectively, and values below 1 in both tumor groups. THY3 was preferentially enriched in non-aggressive tumors (Ro/e = 2.501), whereas THY4, THY5, THY8, THY9, and THY11 were enriched in aggressive tumors. THY5 Ro/e values were 0.021, 0.961, and 1.819 in NT, non-aggressive, and aggressive tissues, respectively.

THY5 cells were detected in 23 of 24 tumor tissue samples, including 13 of 14 aggressive and all 10 non-aggressive tumors. The mean per-sample THY5 fraction among epithelial cells was 14.74% in aggressive tumors and 7.48% in non-aggressive tumors. The sample-level comparison was not statistically significant (two-sided Wilcoxon rank-sum test, P = 0.396). The three largest contributing samples accounted for 84.85% and 84.65% of all THY5 cells in the aggressive and non-aggressive groups, respectively.

Epithelial subpopulations also differed in thyroid differentiation and BRAF- and RAS-related transcriptional features. The thyroid differentiation score (TDS) measures retention of thyroid-specific differentiation programs. TDS was highest in THY1, relatively high in THY6 and THY10, and lowest in THY11. THY5 had an intermediate TDS, below those of the normal-associated THY1 and THY10 populations (Figure 1Q). Conversely, THY5 had a relatively high BRAF-related score, whereas THY1 and THY10 had low scores (Figure 1R). RAS-related scores were highest in THY1 and relatively high in THY10, while THY5 did not show a high score (Figure 1S). Overall differences in TDS, BRAF-related scores, and RAS-related scores were significant across subpopulations (Kruskal–Wallis tests, all P < 2.2 × 10^−16^).

We then examined epithelial subpopulations in relation to lymph node status. The PTC N1 group contained a higher proportion of cells from aggressive tumors (Supplementary Figure S2F). Augur analysis ranked subpopulations by the ability of their transcriptional profiles to distinguish non-aggressive from aggressive disease (Figure 1T). THY2 had the highest AUC (0.791), followed by THY10 (0.760) and THY9 (0.728). THY8 and THY5 also showed discriminatory signals, with AUCs of 0.699 and 0.690, respectively, whereas the remaining subpopulations had lower AUCs. In the comparison of PTC N1 with PTC N0, THY8 ranked first (AUC = 0.850) and THY5 second (AUC = 0.771), followed by THY11 and THY9 (Supplementary Figure S2G).

### 3.2 THY5 and aggressive epithelial states are enriched for extracellular matrix remodeling programs

Marker analysis revealed distinct transcriptional features among the 11 epithelial subpopulations (Figure 2A). THY1 expressed TPO and TFF3, while THY10 was characterized by TFF3 and OTOS. THY2 and THY4 showed SLPI/KRT19 and IGFBP6/PRSS23 expression patterns, respectively. THY8 expressed ISG15 and IFI27, whereas THY11 expressed KRT17 and S100A2. In addition to SFTPB and CXCL14, THY5 showed increased SERPINA1, HLA-DRA, and CD74 expression (Figure 2A). Tissue-group comparisons identified higher TPO, TFF3, and SLC26A4 expression in normal tissues, while aggressive tumors showed increased FN1, S100A4, CTSC, and LGALS1 expression together with reduced TPO, TG, and TFF3 expression (Figure 2B). Gene Ontology (GO) and Kyoto Encyclopedia of Genes and Genomes (KEGG) analyses of THY5-associated genes, together with Reactome enrichment across epithelial subpopulations, further characterized its transcriptional programs. THY5-associated genes were enriched in antigen processing and presentation, T-cell activation and cytotoxicity regulation, major histocompatibility complex (MHC) protein complexes, collagen-containing extracellular matrix, cell adhesion, translation, and insulin-like growth factor-related pathways (Figures 2C–G). Hallmark analysis identified significant enrichment of TNFA_SIGNALING_VIA_NFKB among THY5 upregulated genes (P < 0.01), alongside INFLAMMATORY_RESPONSE, INTERFERON_GAMMA_RESPONSE, INTERFERON_ALPHA_RESPONSE, EPITHELIAL_MESENCHYMAL_TRANSITION, and ANGIOGENESIS (Figure 2H).

**FIGURE 2:**
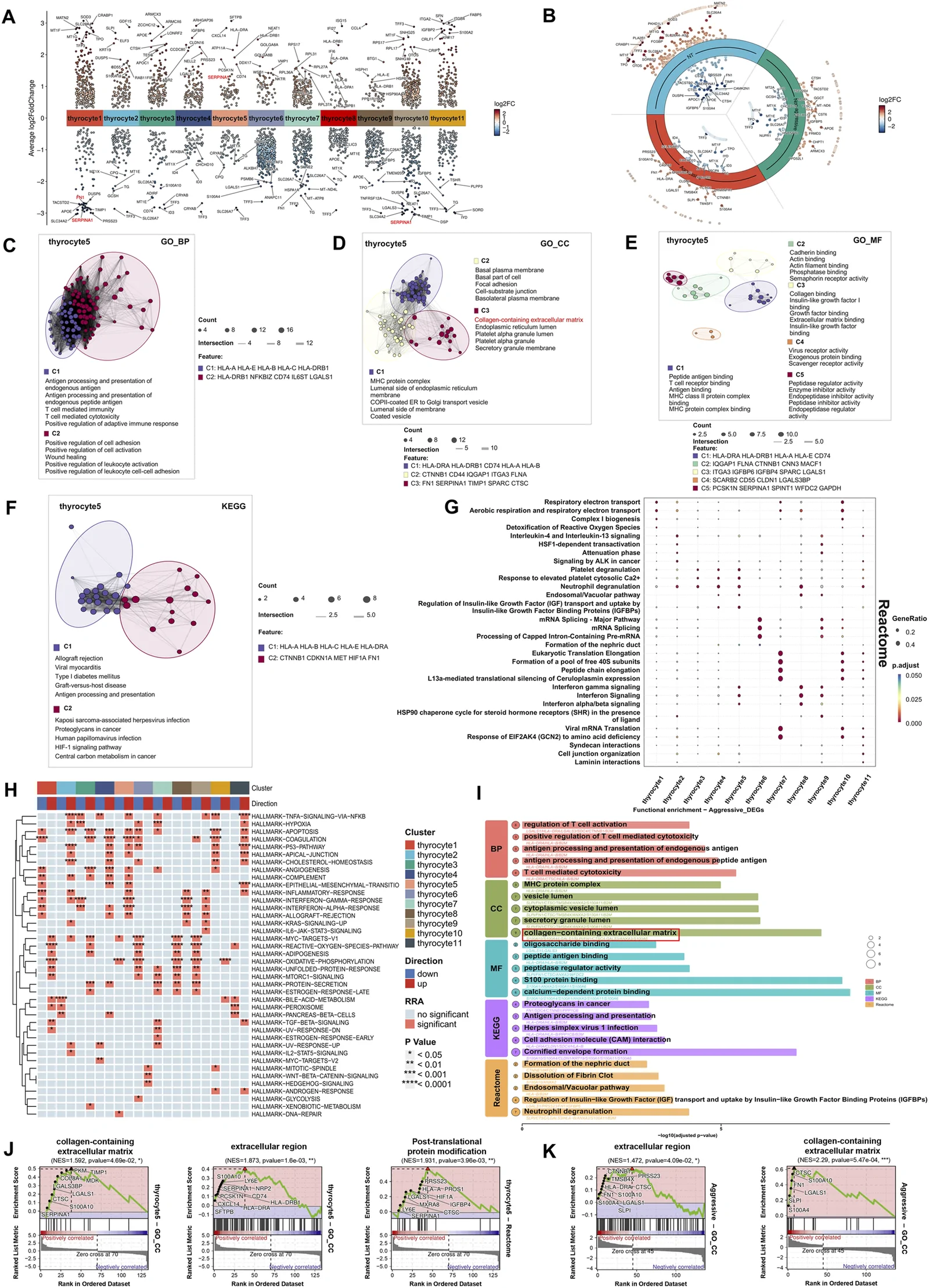
Differential gene expression and pathway enrichment in epithelial subpopulations and tissue groups. **(A)** Marker-gene expression across THY1–THY11. **(B)** Differential-expression patterns by tissue group. **(C–F)** GO biological process, cellular component, molecular function, and KEGG enrichment networks for THY5-associated genes, respectively. **(G)** Reactome enrichment across epithelial subpopulations. **(H)** Hallmark enrichment of upregulated and downregulated gene sets across epithelial subpopulations. **(I)** Enrichment analysis of aggressive-group-associated genes. **(J, K)** GSEA of THY5 and the aggressive group, respectively. GO, Gene Ontology; KEGG, Kyoto Encyclopedia of Genes and Genomes; GSEA, gene set enrichment analysis.

Differentially expressed genes in the aggressive group were enriched in antigen processing and presentation of endogenous peptide antigen, collagen-containing extracellular matrix, cell adhesion molecule (CAM) interaction, and proteoglycans in cancer (Figure 2I). The non-aggressive group showed enrichment in positive regulation of apoptotic signaling, regulation of peptidase activity, platelet alpha granules, collagen-containing extracellular matrix, and post-translational protein phosphorylation (Supplementary Figure S2H). By lymph node status, PTC N0-associated genes were enriched in negative regulation of hydrolase activity and proteolysis, wound healing, coat protein complex II (COPII)-coated endoplasmic-reticulum-to-Golgi transport vesicles, phagocytic vesicles, and antigen presentation involving folding, assembly, and peptide loading of class I MHC (Supplementary Figure S2I). In contrast, PTC N1-associated genes were enriched in collagen-containing extracellular matrix, cell adhesion mediator activity, cadherin binding involved in cell–cell adhesion, integrin signaling, proteoglycans in cancer, wound healing, and post-translational protein phosphorylation (Supplementary Figure S2J).

GSEA showed positive enrichment of collagen-containing extracellular matrix and extracellular region gene sets in both THY5 and the aggressive group; THY5 also showed positive enrichment of post-translational protein modification (Figures 2J,K). PTC N1 similarly showed positive enrichment of collagen-containing extracellular matrix (normalized enrichment score [NES] = 2.428, P = 3.77 × 10^−5^), extracellular region (NES = 1.500, P = 0.0152), post-translational protein phosphorylation (NES = 1.947, P = 0.0056), and extracellular matrix organization (NES = 1.640, P = 0.0367; Supplementary Figures S2K–N).

### 3.3 Trajectory analysis links THY5 to a BRAF-associated epithelial state with reduced differentiation features

PAGA connected THY1 to THY10 and THY6 and also connected THY5 to THY8 (Supplementary Figure S3A). Pseudotime ordering placed THY1 and THY10 predominantly in early regions and THY5 and THY8 in later regions, with THY5 having the second-highest median pseudotime after THY8. Pseudotime distributions differed significantly across subpopulations (Kruskal–Wallis test, P < 2.2 × 10^−16^) (Supplementary Figures S3B–D). Both non-aggressive and aggressive tumor-derived epithelial cells had higher pseudotime than normal cells, with a further increase in the aggressive group (Supplementary Figure S3E). In the nodal grouping, both the combined PTC N0/N1a group (labeled PTC_N0_N1a) and the PTC N1b group (labeled PTC_N1b) had higher pseudotime than normal tissues, but the two PTC groups did not differ significantly (Supplementary Figure S3F). BRAF-related scores correlated positively with pseudotime (R = 0.73), whereas RAS-related scores and TDS correlated negatively (R = −0.56 and −0.63, respectively; all P < 2.2 × 10^−16^; Supplementary Figures S3G–I).

### 3.4 Bulk transcriptomic cohorts support the clinical and microenvironmental associations of THY5

We further subdivided endothelial-associated populations into LECs (LYVE1, PROX1, and CCL21), vascular endothelial cells (VECs; SEMA3G, DLL4, and RGCC), and mural cells (MCs; RGS5, PDGFRB, and TAGLN) according to marker expression (Figure 3A). Their UMAP distributions provided an annotation framework for subsequent deconvolution and communication analyses (Figure 3B). In both TCGA and GEO cohorts, deconvolution showed higher estimated THY5 abundance in tumors, whereas the normal-like reference population THY1 was more abundant in normal tissues (Figure 3C). Estimated THY5 abundance was higher in N1 than in N0 tumors in TCGA, and this association was reproduced in GEO. Later overall stages and higher T stages generally showed greater THY5 abundance, although not all pairwise stage comparisons were significant (Figure 3D).

**FIGURE 3:**
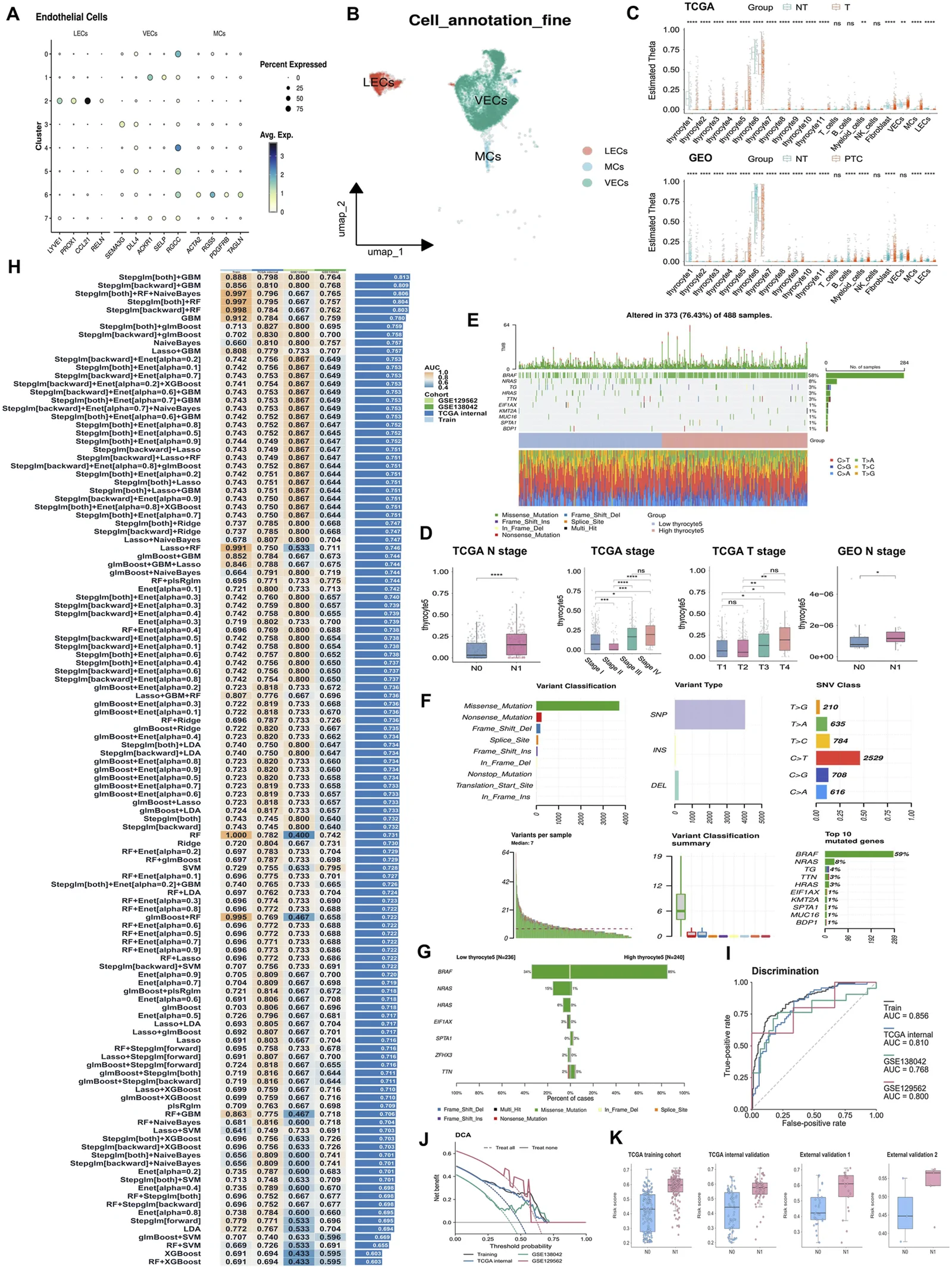
Bulk transcriptomic analyses and exploratory evaluation of the LNM classifier. **(A)** Marker expression for lymphatic endothelial cells (LECs), vascular endothelial cells (VECs), and mural cells (MCs). **(B)** UMAP distributions of these populations. **(C)** Estimated cell-population abundance in tumor and normal tissues from TCGA and GEO. **(D)** THY5 abundance by lymph node status, overall stage, and T stage. **(E)** TCGA oncoplot with THY5-high/low group annotations. **(F)** Summary of variant classes, variant types, single-nucleotide variant classes, and mutation frequencies. **(G)** Mutation frequencies in THY5-high and THY5-low groups. **(H)** Performance comparison of machine-learning algorithm combinations. **(I)** ROC curves of the Stepglm [backward] + GBM classifier in the TCGA training set, TCGA internal evaluation set, GSE138042, and GSE129562. **(J)** Decision curve analyses of the GBM classifier. **(K)** Model-score distributions by N0/N1 status. Evaluation cohorts contributed to model development decisions; the displayed performance is exploratory. UMAP, uniform manifold approximation and projection; ROC, receiver operating characteristic; AUC, area under the curve; GBM, gradient boosting machine; LNM, lymph node metastasis; TCGA, The Cancer Genome Atlas; GEO, Gene Expression Omnibus.

THY5 enrichment was accompanied by a distinct BRAF-related mutational background. BRAF was the most frequently mutated gene in TCGA (Figures 3E,F). BRAF mutation frequency was 85% in the THY5-high group, compared with 34% in the THY5-low group. Conversely, NRAS and HRAS mutation frequencies decreased from 15% to 6% in the THY5-low group to 1% and 0% in the THY5-high group, respectively (Figure 3G). Transcriptional profiling further showed reduced THY1- and THY10-associated signals and increased stromal and immune features in the THY5-high group (Supplementary Figure S3J).

In both TCGA and GEO, the THY5-high group had higher cancer-associated fibroblast (CAF), myeloid-derived suppressor cell (MDSC), and immune exclusion scores, together with higher CD274, IFNG, and cytotoxic T lymphocyte (CTL)-related measures (Supplementary Figure S3L). Drug-response predictions also differed by THY5 status: the THY5-high group had lower predicted values for trametinib but higher values for dabrafenib and sorafenib in both cohorts (Supplementary Figure S3K).

### 3.5 An exploratory 11-gene classifier discriminates lymph node status across cohorts

To assess the value of tumor-associated epithelial transcriptional features for discriminating lymph node status, we integrated WGCNA modules associated with PTC status and estimated THY5 abundance with shared upregulated genes from four bulk comparisons, as detailed in Section 2.5, for feature selection and machine-learning modeling. Among 121 algorithm combinations, backward stepwise regression followed by GBM (Stepglm [backward] + GBM) was selected for the final model (Figure 3H). The model included 11 genes: SCEL, B3GNT3, FN1, CHI3L1, PLCD3, CST6, TIMP1, ADORA1, PRSS23, SERPINA1, and CDH6. AUCs were 0.856 in the training cohort, 0.810 in the TCGA internal evaluation cohort, 0.768 in GSE138042, and 0.800 in GSE129562; the mean AUC across the three evaluation cohorts was 0.793 (Figure 3I). DCA using the same GBM probabilities showed greater estimated net benefit than treat-all and treat-none strategies over threshold probabilities of 0.20–0.70 in TCGA internal evaluation and 0.35–0.71 in GSE138042 (Figure 3J). The eight-sample GSE129562 cohort also showed estimated net benefit over some threshold ranges in this exploratory analysis. Model scores were generally higher in N1 than in N0 tumors across all four cohorts (Figure 3K).

### 3.6 Communication analyses suggest FN1-related interactions between THY5 and LECs

NicheNet analysis with LECs as receiver cells revealed heterogeneous expression changes in candidate upstream ligands across sender populations. In the tumor-versus-NT comparison, FN1 was detected in B cells, some epithelial populations, and LECs, with relative increases in selected cell types (Figure 4A). FN1 ranked 80th in the sender-agnostic analysis and moved to 43rd in the focused analysis, which restricted candidate ligands to those expressed in the predefined sender populations (Figure 4B). In the overall CellChat analysis, ECM–receptor networks showed extensive predicted connections among epithelial, endothelial, and other microenvironmental populations (Figures 4D,E). Outgoing and incoming pathway patterns further revealed epithelial heterogeneity: FN1 was a prominent outgoing signal in THY5, which also received FN1-, COLLAGEN-, and LAMININ-related signals. In contrast, the provisionally normal-like THY1 and THY10 populations showed lower overall predicted activity in these pathways (Supplementary Figure S4A).

**FIGURE 4:**
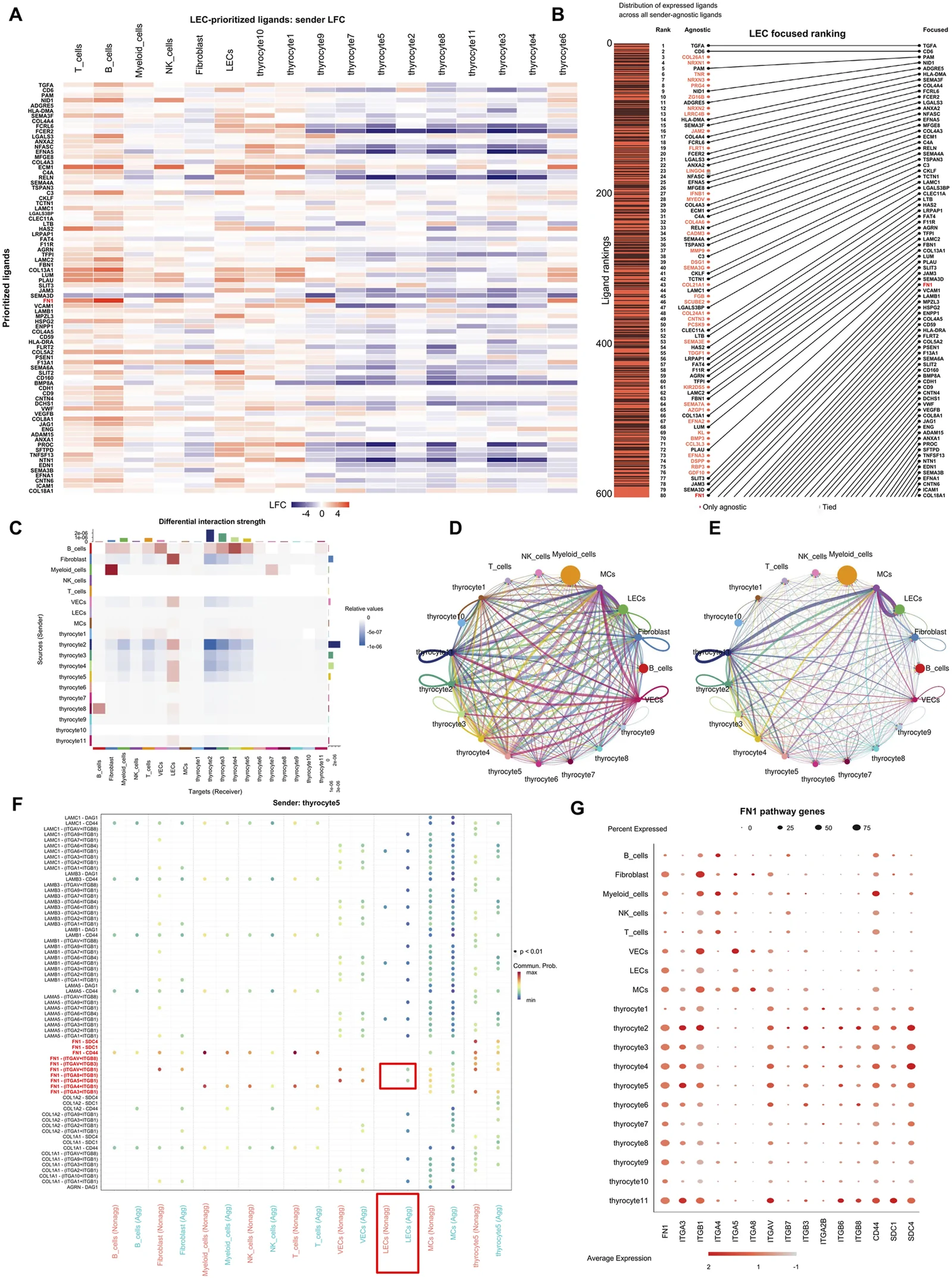
NicheNet and CellChat analyses of epithelial–lymphatic communication. **(A)** Candidate-ligand expression changes across sender populations in the tumor-versus-NT comparison, with LECs as NicheNet receivers. **(B)** Ligand prioritization in sender-agnostic and sender-focused analyses. **(C)** Differences in predicted cell-pair interaction strength between aggressive and non-aggressive groups. **(D, E)** Overall ECM–receptor communication networks. **(F)** Predicted ECM ligand–receptor interactions from THY5 to receiver populations, analyzed separately by clinical group, with THY5-to-LEC FN1–integrin interactions outlined. P values in panel F refer to inferred communication within each group. **(G)** FN1-pathway ligand and receptor gene expression across cell populations; dot size indicates the percentage of expressing cells and color indicates average expression. NT, normal thyroid; LECs, lymphatic endothelial cells; ECM, extracellular matrix.

Restricting the overall network to LEC senders and epithelial receivers identified predicted FN1–SDC4, FN1–CD44, FN1–(ITGAV+ITGB1), and FN1–(ITGA3+ITGB1) interactions between LECs and THY5, with FN1–SDC4 showing a relatively high predicted communication probability. LAMA4 interactions with CD44 or integrin receptors were also detected (Supplementary Figure S4B). Conversely, THY5 formed predicted FN1-related connections with LECs and other microenvironmental populations, as well as multiple epithelial subpopulations, including THY5 itself (Supplementary Figures S4C,D). FN1 pathway heatmaps and networks illustrated the distribution of these interactions across epithelial and microenvironmental populations (Supplementary Figures S4E–I).

To assess aggressiveness-related features of these interactions, we constructed separate CellChat networks for aggressive and non-aggressive cells and compared predicted interaction strengths across cell pairs (Figure 4C). With THY5 fixed as the sender and LECs as the receiver, FN1–(ITGAV+ITGB1) and FN1–(ITGA5+ITGB1) interactions were detected in the aggressive group, both meeting the displayed within-group significance threshold (P < 0.01), whereas the corresponding significant interactions were not displayed in the non-aggressive group (Figure 4F). These receptor complexes correspond to integrins αVβ1 and α5β1. The displayed P values indicate the significance of inferred communication within each group.

Expression plots showed FN1 expression in THY5 and expression of ITGAV, ITGA5, and their shared receptor subunit ITGB1 in LECs (Figure 4G).

### 3.7 Spatial transcriptomics reveals heterogeneous cellular composition and ECM communication in tumors

To characterize aggressive epithelial states and their spatial relationship with LECs, we integrated spatial transcriptomic data from 13 sections of normal thyroid, PTC, and locally advanced PTC (LPTC). We assessed these tissues using spatial clustering, CytoTRACE2, RCTD deconvolution, MISTy neighborhood modeling, ISCHIA co-occurrence analysis, spatial communication inference, and transcriptional regulatory analysis.

Integrated spatial transcriptomic profiles resolved multiple spot-level clusters whose compositions differed across samples (Figures 5A–C). Epithelial–mesenchymal transition (EMT) was particularly prominent in LPTC, together with coagulation, apoptosis, and tumor necrosis factor α (TNF-α)/NF-κB-related programs. PTC showed stronger coagulation, apoptosis, p53, and complement-related programs (Figure 5D). CytoTRACE2 identified differences in inferred developmental potential across spatial transcriptional states, with selected regions exhibiting relatively higher potential (Figure 5E).

**FIGURE 5:**
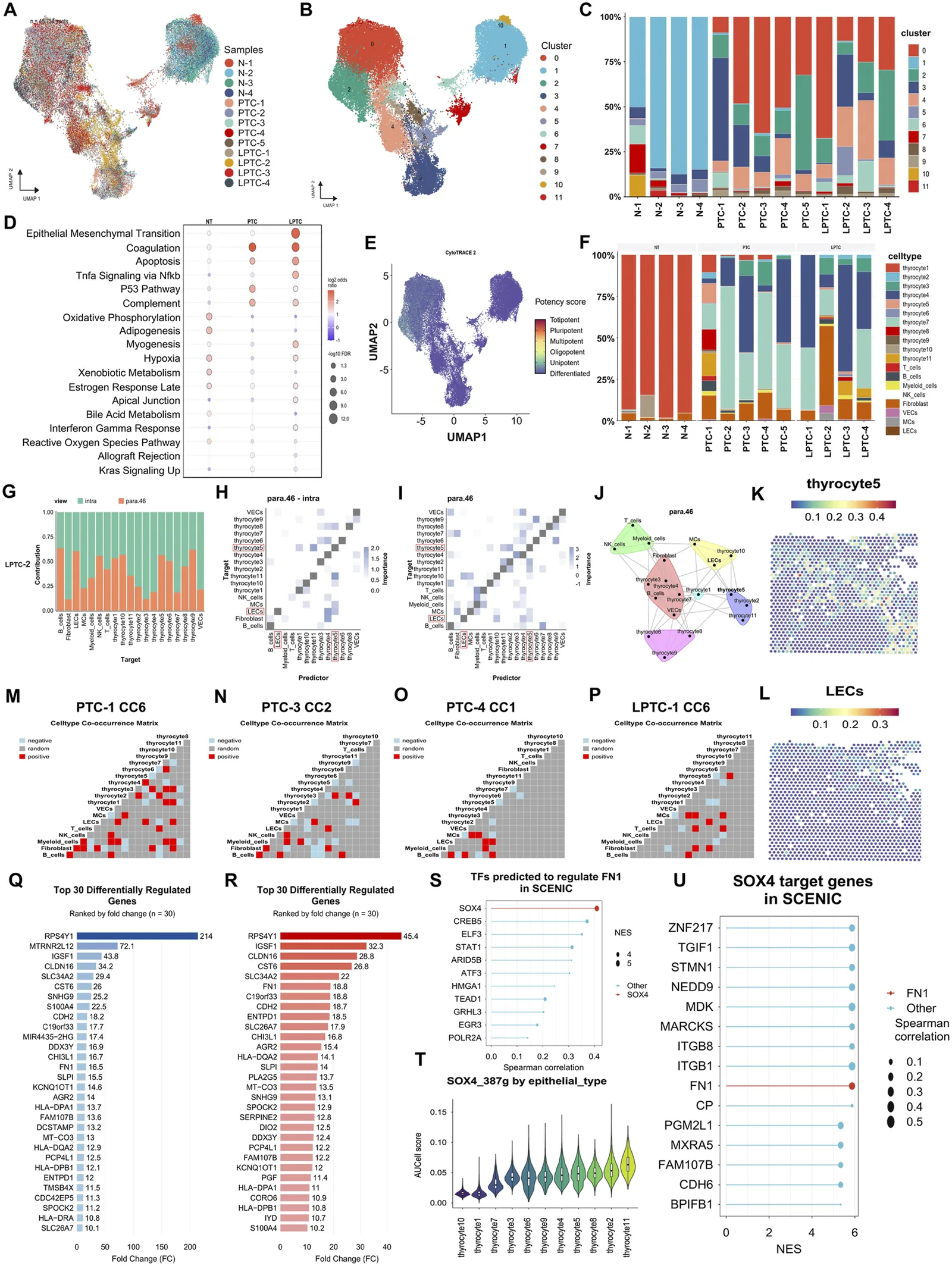
Spatial transcriptomic, neighborhood, co-occurrence, and regulatory analyses. **(A, B)** Integrated spatial-transcriptomic UMAP colored by sample and cluster, respectively. **(C)** Cluster proportions across normal thyroid, PTC, and locally advanced PTC (LPTC) sections. **(D)** Pathway enrichment by tissue group. **(E)** CytoTRACE2 developmental-potential profiles. **(F)** RCTD-derived cell-type compositions. **(G–J)** MISTy neighborhood analyses in the representative LPTC-2 section. **(K, L)** Spatial distributions of THY5 and LEC signals in LPTC-2. **(M–P)** ISCHIA co-occurrence analyses in PTC-1 CC6, PTC-3 CC2, PTC-4 CC1, and LPTC-1 CC6. **(Q, R)** The 30 highest-ranked genes by differential-regulation FC score following SOX4 virtual knockout and overexpression, respectively. FC is a network perturbation score derived from alignment distances. **(S)** FN1-associated candidate transcription factors from SCENIC, plotted by expression correlation with FN1. **(T)** SOX4_387g regulon activity across epithelial subpopulations. **(U)** Predicted SOX4 target genes plotted by normalized enrichment score, with dot size indicating expression correlation. PTC, papillary thyroid carcinoma; LEC, lymphatic endothelial cell; RCTD, robust cell type decomposition; UMAP, uniform manifold approximation and projection; CC, composition cluster; SCENIC, single-cell regulatory network inference and clustering.

RCTD deconvolution further revealed differences in cellular composition across samples (Figure 5F). Normal thyroid was predominantly composed of THY1, with detectable THY10 contributions in some normal samples. PTC and LPTC instead contained heterogeneous mixtures of epithelial subpopulations and stromal cells. THY4 and THY7 accounted for substantial proportions in most tumors, while THY3, THY8, and THY11 contributed to varying degrees. THY5 was relatively scarce in normal thyroid but contributed more prominently in selected PTC and LPTC samples. Regions with higher THY5 abundance commonly also contained other epithelial populations, fibroblasts, immune cells, and vascular endothelial cells.

To compare ECM communication between normal thyroid and tumor tissues, we performed SpatialCellChat analysis in 13 spatial transcriptomic samples (4 normal thyroid, 5 PTC and 4 LPTC samples). Within the ECM–receptor category, FN1 signaling ranked first in all PTC and LPTC samples, whereas COLLAGEN, LAMININ and THBS were the leading pathways in normal thyroid samples (Supplementary Figure S5A).

Getis–Ord Gi analysis of incoming ECM–receptor communication identified spatially heterogeneous Hot, Cold and Other regions, including local ECM communication hotspots within tumor tissues (Supplementary Figure S5B). These maps represent communication across the entire ECM–receptor category and are not specific to FN1 signaling.

### 3.8 THY5 and LECs locally co-occur in selected tumor regions

We used MISTy and ISCHIA to assess whether THY5 and LECs occurred within shared local spatial environments. In the representative LPTC-2 section, MISTy identified predictable local relationships among cell-type compositions, involving multiple epithelial, immune, stromal, and endothelial populations (Figures 5G–J). THY5 and LECs showed patchy distributions within this section, with apparent overlap in selected regions (Figures 5K,L).

ISCHIA was then used for spatial composition clustering and co-occurrence analysis (Figures 5M–P). THY5–LEC co-detection exceeded random expectation in four composition clusters (CCs): PTC-1 CC6, PTC-3 CC2, PTC-4 CC1, and LPTC-1 CC6. Supplementary Figures S6A–D map the locations of these positive clusters, while Supplementary Figure S6E shows the distribution of RCTD-derived cell-type proportions across the integrated spatial atlas.

### 3.9 Regulatory networks and virtual perturbations support SOX4 as an FN1-associated candidate regulator

We integrated SCENIC regulatory networks, virtual perturbations, and cohort-level correlations to investigate the transcriptional regulation of THY5. SOX4 showed a relatively high correlation with FN1 expression among candidate regulators, and FN1 was included in the predicted SOX4 target set (Figures 5S,U). SOX4_387g regulon activity differed across epithelial subpopulations, with higher AUCell activity in THY5 than in the normal-like THY1 and THY10 populations (Figure 5T).

FN1 ranked among the 30 genes with the highest differential-regulation scores (reported as FC by the software) after SOX4 virtual knockout (Figure 5Q; Supplementary Figure S7J) and virtual overexpression (Figure 5R; Supplementary Figure S7I). Regulon specificity and activity varied across epithelial subpopulations (Supplementary Figures S7A,B). SOX4 and FN1, as well as FN1 and SERPINA1, were positively correlated in TCGA and GEO cohorts (Supplementary Figures S7C–F). Correlation matrices of the 11 classifier genes also showed positive FN1–SERPINA1 associations in both cohorts (Supplementary Figure S7G, H). Spatial expression maps of SERPINA1, FN1, and SOX4 in normal thyroid, PTC, and LPTC showed their spatial distributions (Supplementary Figures S8A–M).

### 3.10 OGT is associated with SERPINA1 and changes in O-GlcNAc and FN1 abundance

To investigate the protein context linking SERPINA1 to aggressive ECM phenotypes, we examined IP-MS data (Supplementary Table S1). SERPINA1 (P01009) was identified by 43 peptides, including 41 unique peptides, with 66.99% sequence coverage. OGT (O15294) was identified by 52 peptides, all unique, with 45.12% sequence coverage. Given its enzymatic role in O-linked glycosylation, OGT was selected for further validation as a SERPINA1-associated candidate protein. Exogenous co-IP in 293T cells and endogenous co-IP in BCPAP and TPC-1 cells detected SERPINA1 and OGT within the same immunoprecipitated complexes (Supplementary Figures S9A,B). PLA further detected SERPINA1–OGT and SERPINA1/O-GlcNAc proximity signals in both PTC cell lines (Supplementary Figure S9E).

In BCPAP and TPC-1 cells, OGT knockdown significantly reduced global O-GlcNAc levels together with SERPINA1 and FN1 protein expression, whereas OGT overexpression produced the opposite changes. Re-expression of OGT in the OGT-knockdown background restored these molecular measures (Supplementary Figures S9C,D). Quantification of the functional assays showed fewer colonies, lower EdU-positive fractions, reduced wound closure, and fewer Transwell cells after OGT knockdown in both cell lines. OGT overexpression increased these measures relative to control, and OGT re-expression increased them relative to OGT knockdown alone (Supplementary Figures S9F and S10A).

### 3.11 SOX4 contributes to SERPINA1-associated FN1 expression

In both BCPAP and TPC-1 cells, SERPINA1 knockdown significantly reduced SOX4 and FN1 protein expression (Figures 6A,B). IF showed concurrent reductions in SERPINA1 and FN1 fluorescence intensities (Figures 6C,D). SOX4 knockdown reduced FN1 protein and mRNA levels, whereas SOX4 overexpression increased FN1 mRNA levels (Figures 6E,F,I). ChIP-qPCR showed significant enrichment of the FN1 promoter region in anti-SOX4 immunoprecipitates relative to the IgG control (Figure 6I).

**FIGURE 6:**
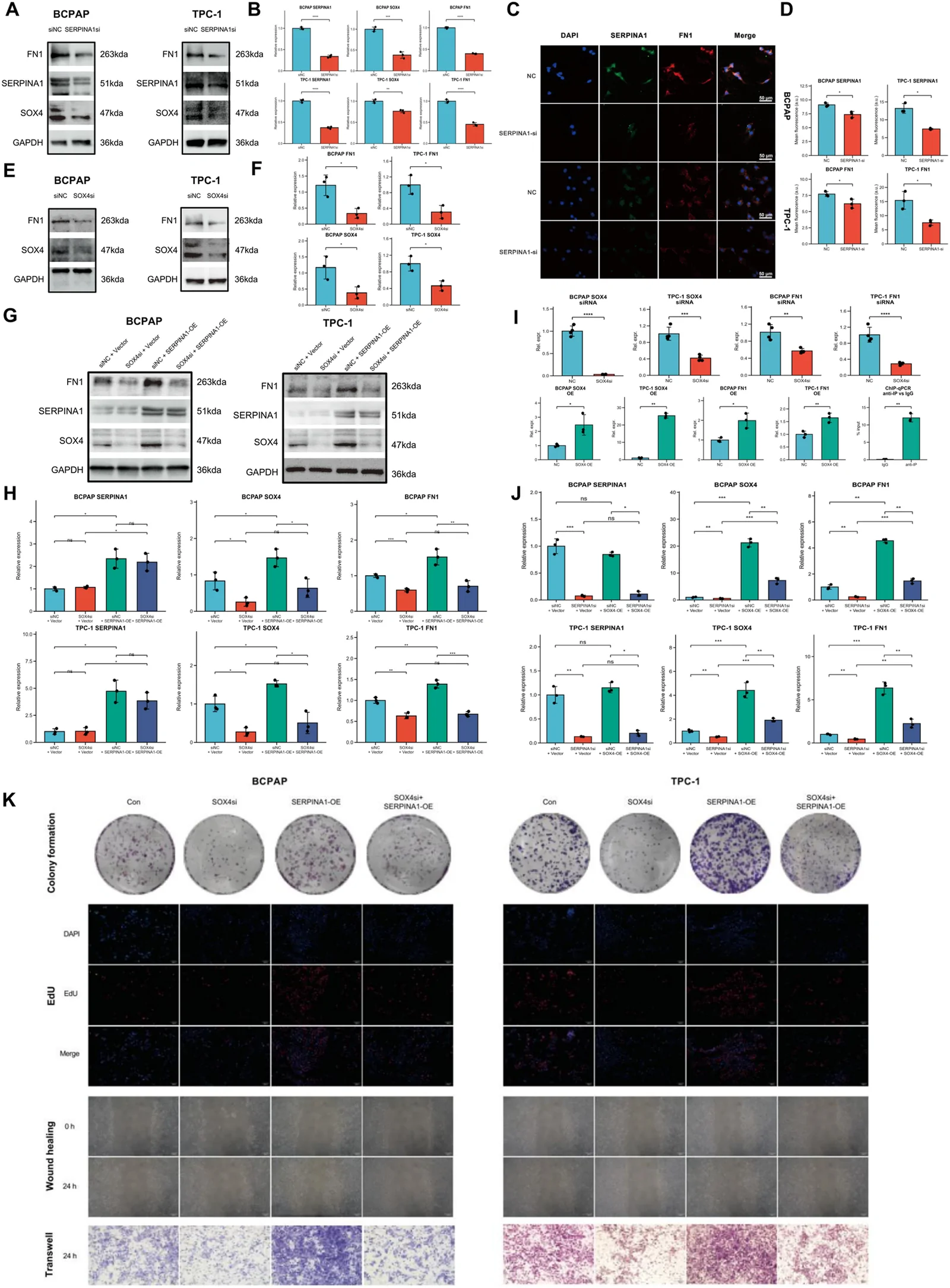
Molecular and functional assays following SERPINA1 and SOX4 perturbation in PTC cells. **(A, B)** Western blots and quantification of SERPINA1, SOX4, and FN1 after SERPINA1 knockdown in BCPAP and TPC-1 cells. **(C, D)** SERPINA1 and FN1 immunofluorescence images and fluorescence quantification after SERPINA1 knockdown; nuclei are stained with DAPI. Scale bars, 50 μm. **(E, F)** Western blots and quantification of SOX4 and FN1 after SOX4 knockdown. **(G, H)** Western blots and quantification of SERPINA1, SOX4, and FN1 under control, SOX4 knockdown, SERPINA1 overexpression, and combined perturbation conditions. **(I)** SOX4 and FN1 mRNA measurements after SOX4 knockdown or overexpression in BCPAP and TPC-1 cells, and ChIP-qPCR analysis of the assayed FN1 promoter region in anti-SOX4 immunoprecipitates and IgG controls. **(J)** SERPINA1, SOX4, and FN1 mRNA measurements in BCPAP and TPC-1 cells under control, SERPINA1 knockdown, SOX4 overexpression, and combined SERPINA1 knockdown plus SOX4 overexpression conditions. In panel J, bars show mean ± SD. Two-sided unpaired Welch t-tests were applied to ΔCt values, with Holm correction across four comparisons within each cell line and gene: knockdown versus control, combination versus knockdown, combination versus overexpression, and overexpression versus control. Corresponding protein experiments are provided in Supplementary Figures S10C,D. **(K)** Representative images of colony formation, EdU incorporation, wound-healing (0 and 24 h), and Transwell assays under control, SOX4 knockdown, SERPINA1 overexpression, and combined perturbation conditions; quantitative results are provided in Supplementary Figure S10B. PTC, papillary thyroid carcinoma; DAPI, 4′,6-diamidino-2-phenylindole; EdU, 5-ethynyl-2′-deoxyuridine; IgG, immunoglobulin G; ChIP-qPCR, chromatin immunoprecipitation followed by quantitative polymerase chain reaction; Ct, cycle threshold; SD, standard deviation. Expression RT-qPCR primer sequences and GAPDH reference-gene information are provided in Supplementary Material 1, Section C; the FN1-999 ChIP-qPCR primer pair is provided in Section B.

Combined perturbation experiments showed that SERPINA1 overexpression increased SOX4 and FN1 protein levels. Concurrent SOX4 knockdown attenuated the increase in FN1 induced by SERPINA1 overexpression, while SERPINA1 protein remained elevated (Figures 6G,H).

We next examined whether SOX4 re-expression could restore FN1 expression after SERPINA1 depletion. In BCPAP and TPC-1 cells, SERPINA1 knockdown reduced SERPINA1, SOX4, and FN1 mRNA levels relative to control. SOX4 overexpression alone increased SOX4 and FN1 mRNA levels without a significant change in SERPINA1 mRNA. In the SERPINA1-knockdown background, SOX4 overexpression increased SOX4 and FN1 mRNA levels while SERPINA1 expression remained low; SOX4 and FN1 mRNA levels remained below those in the SOX4-overexpression-only group (Figure 6J). Western blotting was performed under the same four treatment conditions (Supplementary Figures S10C,D). Compared with SERPINA1 knockdown alone, combined SOX4 re-expression increased FN1 protein from 1.33 ± 0.21 to 3.95 ± 0.21 arbitrary units in BCPAP cells and from 0.21 ± 0.16 to 1.87 ± 0.18 arbitrary units in TPC-1 cells (mean ± SD; unadjusted P < 0.001 for both comparisons). SOX4 protein also increased, whereas SERPINA1 protein did not change significantly relative to knockdown alone. FN1 and SOX4 protein levels did not differ significantly between the combined-treatment and SOX4-overexpression-only groups. Cellular phenotypes were not assessed under this SERPINA1-knockdown/SOX4-re-expression combination.

Functional quantification of the experiments in Figure 6K showed that SOX4 knockdown reduced colony formation, EdU incorporation, wound closure, and Transwell cell counts in BCPAP and TPC-1 cells, whereas SERPINA1 overexpression increased these measures. Concurrent SOX4 knockdown attenuated each increase associated with SERPINA1 overexpression (Supplementary Figure S10B). Compared with SOX4 knockdown alone, combined SERPINA1 overexpression increased colony formation and Transwell cell counts in both cell lines and EdU incorporation in TPC-1 cells; however, the increases in BCPAP EdU incorporation and wound closure in either cell line were not significant.

### 3.12 FN1 re-expression counteracts SERPINA1-knockdown-associated changes in LECs

Compared with controls, thyroid cancer cell-derived CM and co-culture significantly increased FLT4 and PROX1 protein expression in LECs, with larger increases under co-culture. Total NF-κB p65 protein increased under both conditions, while co-culture also significantly increased phosphorylated NF-κB p65 (Figures 7A,B). Quantification of the corresponding functional assays showed increased relative tube formation, Transwell migration, and relative wound healing under both CM treatment and co-culture; co-culture produced larger responses than CM treatment for all three readouts (Figure 7C; Supplementary Figure S10E).

**FIGURE 7:**
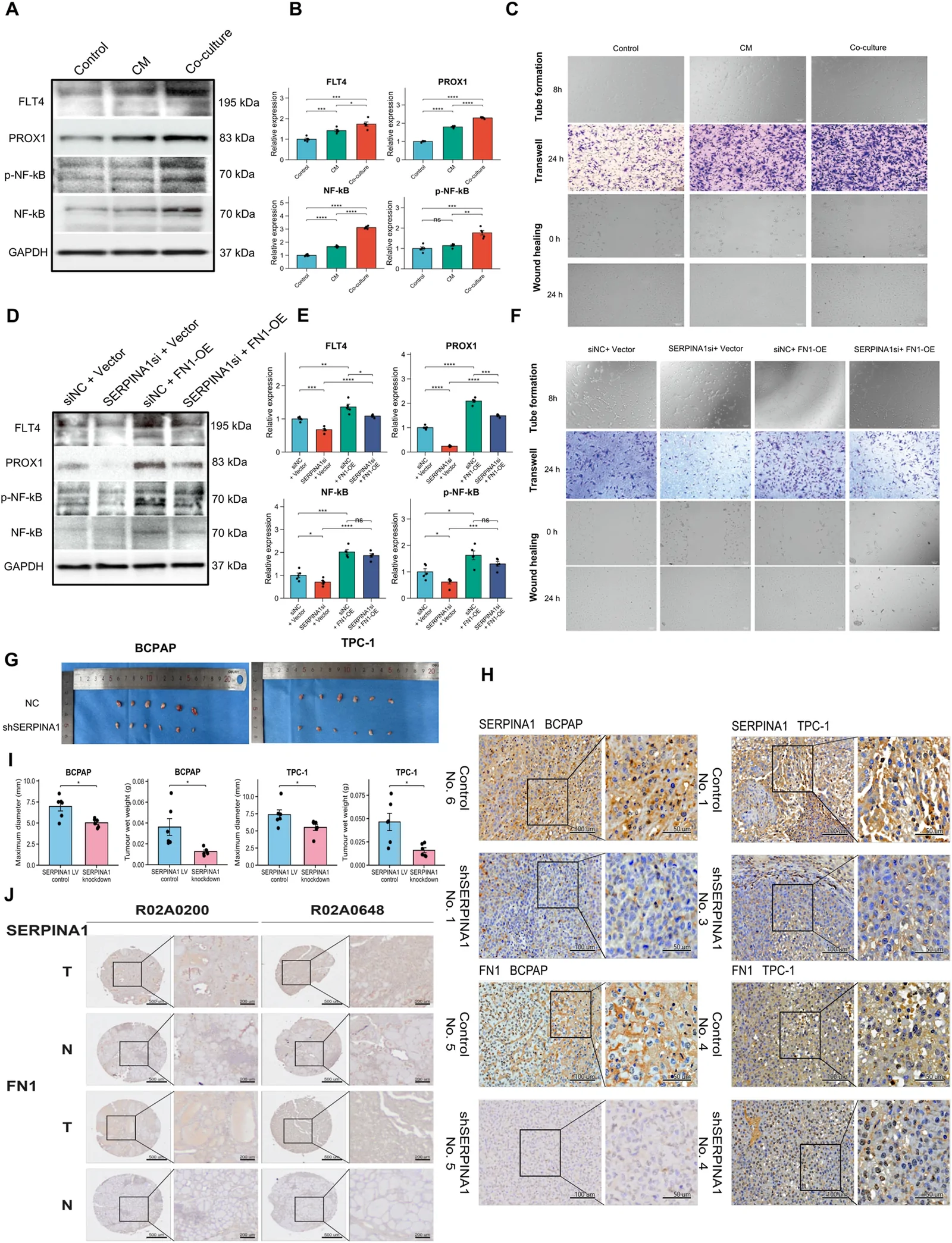
LEC assays, xenograft experiments, and tissue immunohistochemistry. **(A, B)** Western blots and quantification of FLT4, PROX1, total NF-κB p65, and phosphorylated NF-κB p65 in LECs exposed to thyroid cancer cell-conditioned medium or co-culture. **(C)** Representative images of LEC tube-formation, Transwell migration, and wound-healing assays under these conditions; quantitative results are provided in Supplementary Figure S10E. **(D, E)** Western blots and quantification of LEC proteins under tumor-cell control, SERPINA1 knockdown, FN1 overexpression, and combined SERPINA1 knockdown plus FN1 overexpression conditions. **(F)** Representative LEC functional-assay images under these rescue conditions; quantitative results are provided in Supplementary Figure S10E. **(G)** Photographs of excised subcutaneous xenografts from BCPAP and TPC-1 models. **(H)** SERPINA1 and FN1 immunohistochemistry in control and SERPINA1-knockdown xenografts. **(I)** Maximum tumor diameter and wet weight. **(J)** Representative SERPINA1 and FN1 immunohistochemistry in human tumors and corresponding non-tumor thyroid tissues. Tube-formation images were acquired at 8 h, Transwell images at 24 h, and wound-healing images at 0 and 24 h. Scale bars: H, 100 and 50 μm; J, 500 and 200 μm. LEC, lymphatic endothelial cell; CM, conditioned medium; NF-κB, nuclear factor κB; FLT4, fms-related receptor tyrosine kinase 4; PROX1, prospero homeobox 1.

SERPINA1 knockdown in tumor cells significantly reduced FLT4, PROX1, total NF-κB p65, and phosphorylated NF-κB p65 protein levels in recipient LECs. FN1 overexpression in this background significantly increased these molecular measures relative to SERPINA1 knockdown alone (Figures 7D,E). Functional quantification likewise showed reduced relative tube formation, Transwell migration, and relative wound healing after tumor-cell SERPINA1 knockdown. FN1 re-expression significantly increased all three readouts relative to SERPINA1 knockdown alone, although the combined-treatment group remained below the FN1-overexpression-only group (Figure 7F; Supplementary Figure S10E).

### 3.13 Xenograft and human tissue analyses support tumor-associated roles of SERPINA1 and FN1

*In vivo*, SERPINA1-knockdown xenografts derived from BCPAP or TPC-1 cells had significantly smaller maximum tumor diameters and lower tumor weights than controls (Figures 7G,I). IHC showed weaker SERPINA1 and FN1 staining in SERPINA1-knockdown xenografts (Figure 7H). Representative human tissue images also showed stronger SERPINA1 and FN1 staining in tumors than in the corresponding non-tumor tissues (Figure 7J).

Figure 8 distinguishes experimentally supported functional relationships from proposed molecular connections.

**FIGURE 8:**
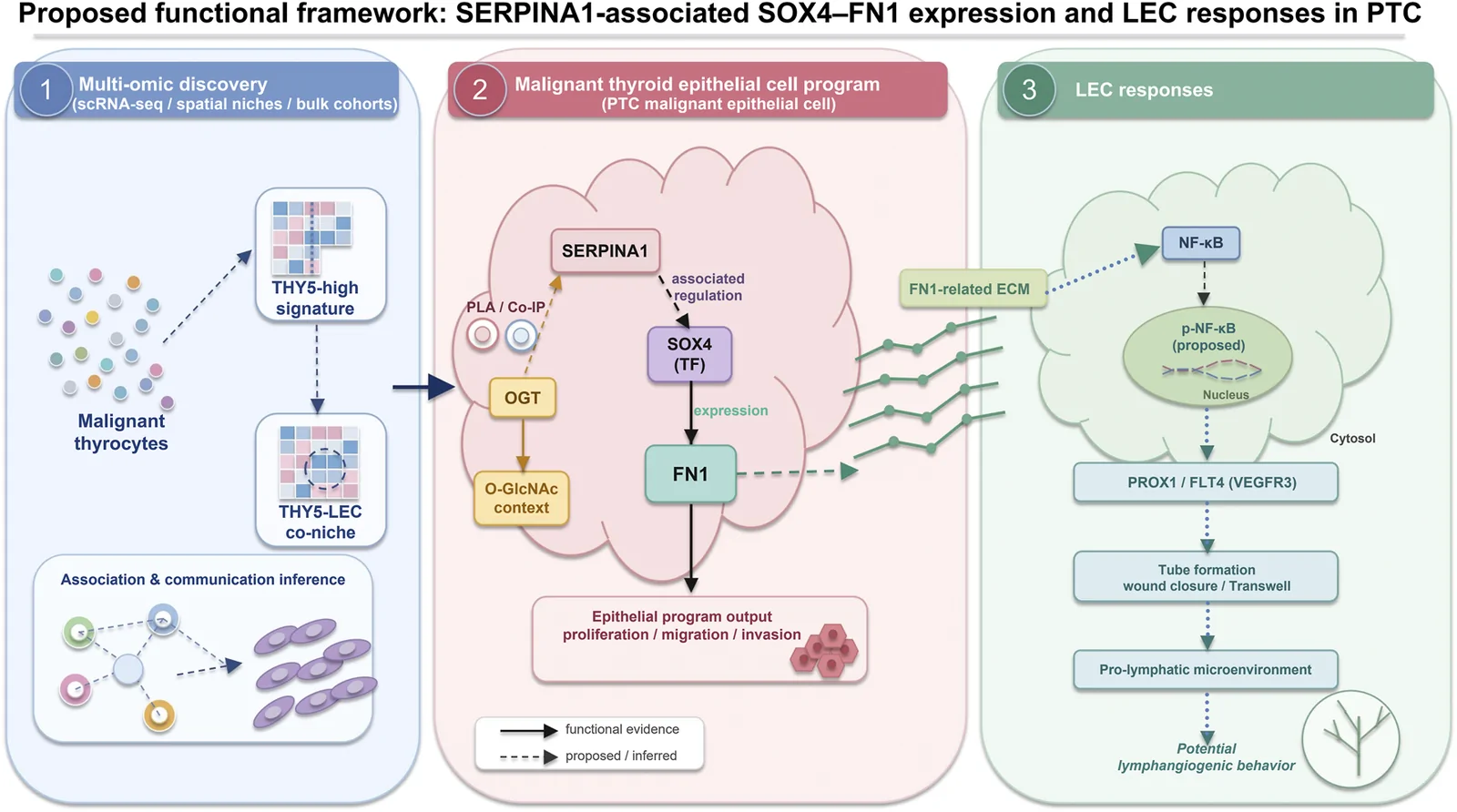
Proposed functional framework linking SERPINA1-associated SOX4–FN1 expression with *in vitro* LEC responses in PTC. The three modules summarize multi-omics associations, the tumor-cell expression program, and proposed LEC responses. Solid arrows denote experimentally supported expression or phenotypic relationships, whereas dashed arrows denote proposed or inferred connections; neither line style establishes a direct molecular interaction. SOX4 perturbation supports FN1 expression changes, and ChIP-qPCR supports SOX4 occupancy at the assayed FN1 promoter region; neither result establishes direct transcriptional regulation. SOX4 re-expression increased FN1 mRNA and protein levels relative to SERPINA1 knockdown alone (Figure 6J; Supplementary Figures S10C,D); phenotypic rescue under this combination remains untested. LEC assays assessed total and phosphorylated NF-κB p65, PROX1 and FLT4/VEGFR3 protein levels and quantified tube-formation and migration responses. The depicted nuclear step, the intervening receptor/signaling mechanisms, and the *in vivo* lymphangiogenic consequences were not established. Direct OGT-mediated modification of SERPINA1 also remains unresolved. PTC, papillary thyroid carcinoma; LEC, lymphatic endothelial cell; ECM, extracellular matrix; TF, transcription factor; OGT, O-linked N-acetylglucosamine transferase; O-GlcNAc, O-linked N-acetylglucosamine; PLA, proximity ligation assay; co-IP, co-immunoprecipitation; scRNA-seq, single-cell RNA sequencing; ChIP-qPCR, chromatin immunoprecipitation followed by quantitative polymerase chain reaction; NF-κB, nuclear factor κB; VEGFR3, vascular endothelial growth factor receptor 3.

## 4 Discussion

LNM remains an important consideration in the clinical assessment and management of generally indolent PTC (Boucai et al., 2024). Epithelial states associated with nodal involvement must therefore be interpreted alongside their interactions with the microenvironment. Our analyses link an aggressiveness-associated epithelial state and its matrix program to experimental evidence relating SERPINA1, SOX4-associated FN1 expression and LEC responses.

Single-cell and spatial studies link thyroid cancer progression to changes in epithelial differentiation and the stromal environment. In PTC, Pu et al. identified follicular-like, partial-EMT-like and dedifferentiation-like thyrocytes. Their dedifferentiation-dominant BRAF-like-B subtype showed low thyroid differentiation and CAF enrichment (Pu et al., 2021). THY5’s high BRAF-related score and lower differentiation relative to normal epithelial references parallel these features. The comparison links BRAF-associated transcription and reduced differentiation to a stromal environment that may support aggressive behavior. In anaplastic transformation, mesenchymal anaplastic thyroid carcinoma (ATC) cells produced abundant collagen and collagen-interacting receptors and interacted strongly with CAFs (Lu et al., 2023). Enrichment of collagen-containing extracellular matrix, extracellular region and ECM organization in our study likewise implicates matrix remodeling across these thyroid cancer contexts. Liao et al. reported high SERPINA1 expression in PTC spatial profiles. They also found FN1 upregulation in the PTC-versus-paratumor ligand comparison (Liao et al., 2025). Those spot-level observations provide tissue context for our epithelial expression analyses. Our tumor-cell perturbation and rescue experiments further connect SERPINA1/FN1 expression to LEC responses.

Preoperative lymph node assessment guides risk stratification and surgical planning in PTC. The 2025 American Thyroid Association guidelines for adult differentiated thyroid cancer highlight limitations in preoperative identification of central neck lymph nodes (Ringel et al., 2025). Those limitations motivate research into molecular information that could complement routine imaging. Our 11-gene LNM classifier linked tumor transcriptional features to N0/N1 status and showed discrimination in the TCGA internal evaluation set and two GEO evaluation cohorts. DCA indicated estimated net benefit over selected threshold ranges in these cohorts. The inclusion of SERPINA1 and FN1 provides an exploratory link between the epithelial and matrix-related functional observations and a clinically relevant nodal phenotype. Its potential value for preoperative assessment requires validation in independent cohorts and samples appropriate to that setting.

SERPINA1 is associated with PTC, but its effects depend on clinical and molecular context. Microarray analysis and independent RT-qPCR identified SERPINA1 as a discriminatory mRNA marker in PTC, with pathway analysis implicating extracellular-matrix interactions (Vierlinger et al., 2011). Li Q. et al. linked SERPINA1 expression to clinicopathological and immune-infiltration features. They also found that its depletion reduced PTC-cell proliferation and migration (Li et al., 2023). Li J. et al. associated SERPINA1 hypomethylation with increased expression in PTC. They reported a functional relationship with MAPK6-associated AKT/mechanistic target of rapamycin (mTOR) signaling (Li J. et al., 2025). An opposing prognostic association was reported in PTC with Hashimoto’s thyroiditis, although the thyroiditis-stratified survival comparison was nonsignificant (Zhang et al., 2023). Our perturbation data extend this functional evidence to FN1-associated tumor-cell and LEC responses. Reduced subcutaneous tumor growth after SERPINA1 depletion further supports its contribution to tumor growth. Enrichment of post-translational protein modification in THY5 motivated investigation of SERPINA1 regulation at the protein level. Reviews of cellular physiology, pathology and multiple cancers describe how O-GlcNAcylation regulates protein function and cancer-associated processes (Chatham et al., 2021; Liu et al., 2024). In PTC, OGT-mediated O-GlcNAcylation of Yes-associated protein (YAP) at Ser109 promoted YAP activation and malignant cellular phenotypes (Li et al., 2021). Elevated O-GlcNAcylation also enhanced proliferation, colony formation, migration and invasion in ATC (Cheng et al., 2016). Our IP-MS screen identified OGT as a candidate SERPINA1-associated protein, prompting investigation of OGT and O-GlcNAcylation in SERPINA1 regulation. Both co-IP and PLA supported this association, and OGT perturbation altered SERPINA1/FN1 abundance. These observations connect thyroid-cancer O-GlcNAc regulation with the SERPINA1–FN1 functional relationship and motivate further study of post-translational regulation of epithelial matrix-related programs.

FN1 may connect epithelial transcriptional programs to matrix-dependent cellular behavior. Across solid tumors including breast, pancreatic and lung cancer, biochemical, mechanical and architectural changes in the matrix shape progression and dissemination (Cox, 2021). In PTC, elevated FN1 was associated with nodal disease and extrathyroidal extension. FN1 loss or antagonism of fibronectin–αvβ3 interactions reduced proliferation and migration (Rocco et al., 2025). Jiang et al. identified FN1 as a target of miR-142–3p in PTC. They linked FN1 perturbation to focal adhesion kinase (FAK)/extracellular signal-regulated kinase (ERK)/phosphoinositide 3-kinase (PI3K) signaling and malignant cellular behaviors (Jiang et al., 2023). However, the effects of FN1 vary with its molecular form. Intracellular extra domain B (EDB)-negative FN1 restrained PTC-cell proliferation through p21-associated effects. EDB-positive FN1 instead favored invasion, endothelial tube formation and nodal metastasis in those models (Liu et al., 2025). Their conditioned-medium experiments in LECs and vascular endothelial cells are particularly relevant to our study. These divergent isoform effects indicate that total FN1 abundance does not fully capture its biological activity. Our perturbation and rescue experiments connect tumor-cell SERPINA1/FN1 expression to LEC protein and cellular responses. Quantified tube-formation, Transwell and wound-healing assays showed that FN1 re-expression counteracted the effects of SERPINA1 depletion in PTC cells on recipient LECs. These results support FN1 involvement in SERPINA1-associated LEC responses, alongside its association with malignant tumor-cell behavior. This functional evidence supports further investigation of matrix-associated epithelial–lymphatic interactions.

SOX4 perturbation supports a functional contribution to SERPINA1-associated FN1 expression. In the PTC atlas of Pu et al., SOX4 was among the transcription factors associated with the dedifferentiation-like thyrocyte program (Pu et al., 2021). In differentiated thyroid cancer, SOX4 depletion reduced cell growth, clonogenicity and anoikis resistance. These effects accompanied changes in AKT/ERK phosphorylation, CRABP2 and integrin β1/β4 expression (Kuo et al., 2023). In PTC, circ_0039411 regulated the miR-423–5p/SOX4 relationship, and SOX4 overexpression reversed several effects of circ_0039411 depletion (Wen et al., 2021). SOX4 thus regulates thyroid-cell behavior while itself receiving multiple regulatory inputs. In normal and cancerous breast epithelial models, Sox4 regulated Ezh2 expression and epigenetic reprogramming during EMT (Tiwari et al., 2013). A review across cancers also described SOX4 contributions to migration, invasion, stemness and survival within EMT programs (Lourenço and Coffer, 2017). In our experiments, SOX4 perturbation altered FN1 expression, and ChIP-qPCR supported SOX4 occupancy at the assayed FN1 promoter region. SOX4 re-expression increased FN1 mRNA and protein levels relative to SERPINA1 knockdown alone, extending the mRNA rescue finding to protein expression. The nonsignificant protein-level differences between combined treatment and SOX4 overexpression alone do not establish equivalence. Cellular phenotypes under that same treatment combination remain untested in our study. The separate SOX4-knockdown/SERPINA1-overexpression experiments showed that SOX4 depletion attenuated SERPINA1-associated cellular responses. However, they cannot substitute for phenotypic testing of SOX4 re-expression in SERPINA1-depleted cells. The molecular steps through which SERPINA1 influences SOX4 also remain unresolved.

Lymphangiogenesis and lymphatic remodeling contribute to tumor dissemination across multiple cancer types (Stacker et al., 2014). The FN1-associated LEC responses observed here should be interpreted within this lymphatic microenvironment. In pediatric and young-adult PTC, Guo et al. predicted COLLAGEN-, FN1- and PERIOSTIN-related communication between matrix-associated CAFs and EMT-enriched thyrocyte State 3. Multiplex IHC also associated higher tumor-cell VEGFC expression with VEGFR3-positive lymphatic endothelium (Guo et al., 2025). These findings connect matrix-associated cellular states with lymphatic responses, providing context for our FN1-associated LEC observations. In mouse skin squamous-cell carcinogenesis, vascular endothelial growth factor C (VEGF-C) expanded sentinel-node lymphatic networks before metastasis. It promoted subsequent dissemination without increasing primary tumor growth (Hirakawa et al., 2007). Lymphatic remodeling can therefore contribute to tumor progression beyond its effects on primary tumor size.

The cellular source and signaling route shape LEC responses to tumors. In early-stage bladder cancer, CAF-derived ITGA11 engaged SELE on LECs and SRC–VEGFR3–mitogen-activated protein kinase (MAPK) signaling (Zheng et al., 2024). Our results support an epithelial contribution to matrix-associated LEC responses. FN1 re-expression in SERPINA1-depleted PTC cells increased LEC protein marker abundance and counteracted reductions in quantified tube formation and migration. The pan-cancer vascular atlas of Pan et al. distinguished lymphangiogenic and antigen-presenting LEC trajectories. It proposed an NFKB1–PROX1–VEGFR3 regulatory model for lymphangiogenesis and identified NF-κB-related signaling enrichment in tip-like and stalk-like LECs (Pan et al., 2024). Changes in PROX1, FLT4/VEGFR3 and NF-κB-related proteins in our experiments are consistent with this model. Our data associate those protein changes and LEC behavior with the SERPINA1/FN1 program in PTC tumor cells. In intrahepatic cholangiocarcinoma and hepatocellular carcinoma, tumor-secreted S100A6 increased vascular endothelial growth factor D (VEGF-D) production in human LECs. This response involved receptor for advanced glycation end products (RAGE)/NF-κB signaling (Chen et al., 2024), providing another example of tumor-derived signals engaging NF-κB-associated LEC responses. The consequences of lymphatic responses also depend on the treatment context. In melanoma models, VEGF-C enhanced immunotherapy responses through CCL21-associated recruitment of naïve T cells, and CCR7 blockade reversed this benefit (Fankhauser et al., 2017).

Several limitations should be considered when interpreting these findings. The LNM classifier’s evaluation cohorts contributed to candidate screening and model selection, so its performance estimates may be optimistic. Its findings remain exploratory, particularly in the eight-sample GSE129562 cohort, and require independent validation of a prespecified model and preprocessing pipeline. The aggressive group also combined clinically heterogeneous PTC subgroups in this study. Sample-level THY5 abundance did not differ significantly between tumor groups, and a few samples contributed most THY5 cells, limiting generalization of pooled-cell enrichment. Spatial co-occurrence was restricted to certain tissue contexts, and spot-level resolution cannot establish direct epithelial–LEC contact; multiplex imaging in additional cases is needed. Functional perturbation and mRNA/protein rescue do not establish a direct SERPINA1–SOX4–FN1 regulatory sequence. ChIP-qPCR supports SOX4 occupancy at the assayed FN1 promoter region, but reporter assays and binding-site mutagenesis are needed to establish its regulatory function. The molecular receptors mediating LEC responses also remain undefined in this study. Direct OGT-mediated modification of SERPINA1 requires substrate-specific biochemical and site-level validation. LEC effects were evaluated *in vitro*, whereas the subcutaneous model assessed tumor growth without examining the thyroid microenvironment, lymphangiogenesis or LNM. Models with lymphatic and nodal endpoints are therefore needed to assess relevance to metastasis.

The evidence supports functional relationships among SERPINA1-associated SOX4–FN1 expression, tumor-cell behavior and *in vitro* LEC responses. OGT/O-GlcNAc-associated changes provide an additional molecular context for these functional observations. Further work should resolve molecular connections, test LEC responses and LNM in appropriate *in vivo* models, and evaluate the exploratory classifier in independent clinical cohorts.

## 5 Conclusion

This study supports functional links among the SERPINA1-associated molecular program, malignant epithelial cell behavior and LEC responses in PTC, providing evidence for the involvement of an FN1-associated matrix program in epithelial–lymphatic interactions. These findings broaden the perspective on PTC aggressiveness from changes within tumor cells to epithelial–lymphatic interactions and highlight the potential value of SERPINA1 and its associated molecular network for investigating therapeutic interventions. Meanwhile, a lymph node metastasis classifier incorporating SERPINA1 and FN1 provides an exploratory framework for linking molecular features to clinical lymph node status, highlighting the potential of these findings for molecularly informed risk assessment. Together, this work provides a basis for further investigation of metastasis-related mechanisms and individualized diagnostic and therapeutic strategies in PTC.

## Data Availability

The original contributions presented in the study are included in the article/Supplementary Material, further inquiries can be directed to the corresponding author.
