# Supplementary material for: A SERPINA1-associated SOX4–FN1 epithelial program linked to lymphatic endothelial responses in aggressive papillary thyroid carcinoma
Source: Front Cell Dev Biol. 2026 Oct 27;14:1929991. doi: 10.3389/fcell.2026.1929991 (PMC13748456; doi:10.3389/fcell.2026.1929991)
Supplement: Supplementary file 1 [file Supplementaryfile1.docx]

**Supplementary Materials**

Gene-perturbation oligonucleotides, FN1 ChIP-qPCR primers, expression RT-qPCR primers, and overexpression plasmid maps

# Sequence list

## A. Gene-perturbation oligonucleotides (siRNA and sgRNA cloning oligos)

| **Molecule** | **Type** | **Name / strand** | **Sequence (5'-3')** |
| --- | --- | --- | --- |
| SERPINA1 | siRNA | Forward | GGCUGACACUCACGAUGAATT |
| SERPINA1 | siRNA | Reverse | UUCAUCGUGAGUGUCAGCCTT |
| FN1 | siRNA | Forward | GUGCAGCACAACUUCGAAUTT |
| FN1 | siRNA | Reverse | AUUCGAAGUUGUGCUGCACTT |
| SOX4 | siRNA | Forward | GCGACAAGAUCCCUUUCAUTT |
| SOX4 | siRNA | Reverse | AUGAAAGGGAUCUUGUCGCTT |
| OGT | siRNA | Forward | GAUUAAGCCUGUUGAAGUC |
| OGT | siRNA | Reverse | GACUUCAACAGGCUUAAUC |
| SERPINA1 | sgRNA cloning oligo | Forward | CACCGTGCTGACCATCGACGAGAAA |
| SERPINA1 | sgRNA cloning oligo | Reverse | AAACTTTCTCGTCGATGGTCAGCAC |

The siRNA duplexes and sgRNA cloning oligos below are gene-perturbation reagents; they are not PCR primers. Overexpression plasmid maps are shown after the primer tables.

## B. FN1 promoter ChIP-qPCR primers

| **Target region** | **Forward primer (5'-3')** | **Reverse primer (5'-3')** | **Assay** |
| --- | --- | --- | --- |
| FN1-999 | ATGTGAGAAAGGGGCCGAAG | CAGCTGTGGGGAGAGAAAGG | ChIP-qPCR |

These primers interrogate the FN1 promoter region in ChIP-qPCR; they are not used to quantify FN1 mRNA expression.

## C. Expression RT-qPCR primers and reference gene

| **Gene** | **Forward primer (5'-3')** | **Reverse primer (5'-3')** | **Role** |
| --- | --- | --- | --- |
| SERPINA1 | AAGAGCGTCCTGGGTCAACTG | CGATGGTCAGCACAGCCTTATG | Expression RT-qPCR |
| SOX4 | AAGATCATGGAGCAGTCGCC | CGCCTCTCGAATGAAAGGGA | Expression RT-qPCR |
| GAPDH | CAAATTCCATGGCACCGTCA | GGACTCCACGACGTACTCAG | Reference gene |
| FN1 | CGGTGGCTGTCAGTCAAAG | AAACCTCGGCTTCCTCCATAA | Expression RT-qPCR |

# **OGT overexpression plasmid map**

# **FN1 overexpression plasmid map**

# **SERPINA1 overexpression plasmid map**

**SOX4 overexpression plasmid map**
