# Supplementary material for: A SERPINA1-associated SOX4–FN1 epithelial program linked to lymphatic endothelial responses in aggressive papillary thyroid carcinoma
Source: Front Cell Dev Biol. 2026 Oct 27;14:1929991. doi: 10.3389/fcell.2026.1929991 (PMC13748456; doi:10.3389/fcell.2026.1929991)
Supplement: Supplementary file 2 [file Supplementaryfile2.docx]

**Supplementary Figure 1. Overview of the multi-omics analysis and experimental workflow in PTC.**

The six numbered modules depict (1) thyroid tissue and single-cell, spatial, and bulk transcriptomic resources; (2) epithelial subpopulation analysis, including cell-state, CNV, genomic instability, and cellular composition assessments; (3) differential gene expression and pathway enrichment analyses; (4) ligand–receptor communication and spatial epithelial–lymphatic niche analyses; (5) tumor cell–LEC co-culture, perturbation experiments, and proximity ligation assays; and (6) xenograft experiments and immunohistochemical analyses of xenografts and human tissue microarrays. PTC, papillary thyroid carcinoma; LEC, lymphatic endothelial cell; CNV, copy-number variation; GIS, genomic instability score; DEG, differentially expressed gene; GSEA, gene set enrichment analysis; ECM, extracellular matrix; PLA, proximity ligation assay; IHC, immunohistochemistry; TMA, tissue microarray.

**Supplementary Figure 2. Single-cell annotations, epithelial CNV scores, and phenotype-associated analyses.**

(A, B) UMAP of the integrated single-cell dataset colored by sample and unsupervised cluster, respectively. (C) Dot plot of canonical cell-type markers across clusters; color indicates mean expression and dot size indicates the fraction of expressing cells. (D) UMAP of epithelial cells colored by sample. (E) CNV score distributions across epithelial subpopulations, with an overall Kruskal–Wallis comparison. (F) Proportions of cells from aggressive and non-aggressive tumors within the PTC N0 and PTC N1 groups. (G) Augur analysis of epithelial subpopulations comparing PTC N1 with PTC N0. (H–J) GO, KEGG, and Reactome enrichment analyses of genes associated with non-aggressive tumors, PTC N0, and PTC N1, respectively. (K–N) GSEA plots for collagen-containing extracellular matrix, extracellular region, post-translational protein phosphorylation, and extracellular matrix organization, respectively, in the PTC N1 analysis. The labels PTC_N0 and PTC_N1 in the panels correspond to PTC N0 and PTC N1, respectively. PTC, papillary thyroid carcinoma; UMAP, uniform manifold approximation and projection; CNV, copy-number variation; GO, Gene Ontology; KEGG, Kyoto Encyclopedia of Genes and Genomes; GSEA, gene set enrichment analysis; NES, normalized enrichment score; AUC, area under the curve.

**Supplementary Figure 3. Epithelial trajectory analysis and bulk transcriptomic comparisons by THY5 abundance.**

(A) PAGA representation of relationships among epithelial subpopulations. (B, C) Trajectory embeddings colored by pseudotime and epithelial subpopulation, respectively. (D–F) Pseudotime distributions by epithelial subpopulation, tissue group, and nodal group, respectively. Panel D uses a Kruskal–Wallis test; panels E and F display Wilcoxon comparisons. (G–I) Relationships of pseudotime with BRAF-related, RAS-related, and thyroid differentiation scores, respectively; point color indicates log10(nCount_RNA), where nCount_RNA is the total RNA count per cell. (J) TCGA expression heatmap comparing THY5-high and THY5-low samples, with cell-composition and microenvironmental annotations, adjusted-P-value annotations, and log-fold-change summaries. (K) Predicted responses to dabrafenib, sorafenib, and trametinib in THY5-high and THY5-low groups in TCGA and GEO. (L) CAF, CD274, CTL, immune exclusion, IFNG, and MDSC measures by THY5 abundance in TCGA and GEO. High and low groups were defined using the median estimated THY5 abundance within each cohort. PAGA, partition-based graph abstraction; NT, normal thyroid; TDS, thyroid differentiation score; CAF, cancer-associated fibroblast; CTL, cytotoxic T lymphocyte; MDSC, myeloid-derived suppressor cell; ns, not significant; TCGA, The Cancer Genome Atlas; GEO, Gene Expression Omnibus.

**Supplementary Figure 4. Predicted ECM–receptor and FN1 communication networks inferred by CellChat.**

(A) Predicted outgoing and incoming ECM–receptor signaling patterns across annotated cell populations; heatmap color indicates relative inferred signaling strength. (B–D) Predicted ligand–receptor interaction dot plots for LEC-to-thyrocyte, THY5-to-thyrocyte, and THY5-to-immune/stromal communication, respectively. Dot color represents inferred communication probability and dot size represents statistical significance of the inferred communication. (E) Inferred FN1 signaling heatmap with sender populations in rows and receiver populations in columns. (F, G) Hierarchical representations of the inferred FN1 signaling network showing epithelial and microenvironmental populations, including vascular-associated populations. (H, I) Predicted circular networks for FN1–(ITGA5+ITGB1) and FN1–(ITGAV+ITGB1), respectively. ECM, extracellular matrix; LEC, lymphatic endothelial cell; VEC, vascular endothelial cell; MC, mural cell.

**Supplementary Figure 5. SpatialCellChat analysis of ECM–receptor signaling and spatial communication hotspots.**

(A) ECM–receptor signaling pathways ranked by information flow in each spatial sample. FN1 labels are highlighted in red. (B) Spatial classification of incoming ECM–receptor communication using Getis–Ord Gi analysis; red, blue and gray spots denote Hot, Cold and Other regions, respectively. The maps represent communication across the entire ECM–receptor category and are not specific to FN1 signaling. Both panels include 13 samples: N-1–N-4, PTC-1–PTC-5 and LPTC-1–LPTC-4. N, normal thyroid; PTC, papillary thyroid carcinoma; LPTC, locally advanced papillary thyroid carcinoma; ECM, extracellular matrix.

**Supplementary Figure 6. Spatial composition clusters and cell-type proportion maps.**

(A–D) Composition-cluster maps overlaid on tissue images for PTC-1 (GSM7980864), PTC-3 (GSM7980866), PTC-4 (GSM7980867), and LPTC-1 (GSM7980868), respectively. Spot colors indicate composition clusters CC1–CC7. The panel headings identify CC6, CC2, CC1, and CC6, respectively, for the corresponding THY5–LEC co-occurrence analyses. (E) RCTD-derived proportions of epithelial, immune, fibroblast, vascular endothelial, mural, and lymphatic endothelial populations displayed on the integrated spatial UMAP. Color scales indicate cell-type proportions, and inset histograms show their distributions across spots. CC, composition cluster; RCTD, robust cell type decomposition; LEC, lymphatic endothelial cell; VEC, vascular endothelial cell; MC, mural cell; LPTC, locally advanced papillary thyroid carcinoma; PTC, papillary thyroid carcinoma; UMAP, uniform manifold approximation and projection.

**Supplementary Figure 7. Regulon profiles, gene-expression correlations, and SOX4 virtual perturbation analyses.**

(A) Regulon specificity scores and rankings across THY1–THY11. (B) Heatmap of regulon activity across epithelial subpopulations. (C, D) TCGA scatterplots of SOX4 versus FN1 and FN1 versus SERPINA1 expression, respectively. (E, F) Corresponding expression scatterplots in GEO. Pearson and Spearman correlation analyses are displayed in panels C–F. (G, H) Spearman correlation matrices for the 11 classifier genes in TCGA and GEO, respectively. (I) Differential-regulation plot following SOX4 virtual overexpression. (J) Differential-regulation plot following SOX4 virtual knockout. In panels I and J, the horizontal axis represents the Z-score and the vertical axis represents −log10(q); selected genes are labeled. q denotes the false-discovery-rate-adjusted P value. TCGA, The Cancer Genome Atlas; GEO, Gene Expression Omnibus.

**Supplementary Figure 8. Spatial expression maps of SERPINA1, FN1, and SOX4.**

Each panel shows SERPINA1, FN1, and SOX4 expression from left to right, overlaid on the corresponding tissue image. (A–D) Normal thyroid sections N-1 (GSM7980860), N-2 (GSM7980861), N-3 (GSM7980862), and N-4 (GSM7980863), respectively. (E–I) PTC-1 (GSM7980864), PTC-2 (GSM7980865), PTC-3 (GSM7980866), PTC-4 (GSM7980867), and PTC-5 (GSM7980875), respectively. (J–M) LPTC-1 (GSM7980868), LPTC-2 (GSM7980869), LPTC-3 (GSM7980870), and LPTC-4 (GSM7980871), respectively. Spot colors represent expression levels according to the individual color scale above each map. PTC, papillary thyroid carcinoma; LPTC, locally advanced papillary thyroid carcinoma.

**Supplementary Figure 9. Co-immunoprecipitation, proximity ligation, and OGT perturbation assays.**

(A) Anti-Flag co-immunoprecipitation in 293T cells transfected with Flag-OGT and/or HA-SERPINA1, with input lysates and immunoprecipitates analyzed by western blotting. (B) Endogenous co-immunoprecipitation analysis of OGT and SERPINA1 in BCPAP and TPC-1 cells, including input, IgG, and IP lanes. (C) Western blots for O-GlcNAc (RL2), FN1, OGT, and SERPINA1 in BCPAP and TPC-1 cells under siNC + Vector, OGTsi + Vector, siNC + OGT-OE, and OGTsi + OGT-OE conditions; GAPDH is the loading control. (D) Quantification of the immunoblot signals in panel C. (E) PLA images for SERPINA1–OGT and SERPINA1/O-GlcNAc antibody pairs in BCPAP and TPC-1 cells, with negative controls. DAPI, PLA, and merged images are shown; scale bars, 50 μm. (F) Representative images of colony formation, EdU incorporation, wound-healing, and Transwell assays under the four conditions used in panel C. Wound-healing images were acquired at 0 and 24 h, and Transwell images at 24 h. Quantitative results for panel F are shown in Supplementary Figure 10A. IP, immunoprecipitation; PLA, proximity ligation assay; siNC, negative-control siRNA; OGTsi, OGT-targeting siRNA; OE, overexpression; OGT, O-linked N-acetylglucosamine transferase; O-GlcNAc, O-linked N-acetylglucosamine; EdU, 5-ethynyl-2′-deoxyuridine; DAPI, 4′,6-diamidino-2-phenylindole; GAPDH, glyceraldehyde-3-phosphate dehydrogenase; IgG, immunoglobulin G; siRNA, small interfering RNA; HA, hemagglutinin epitope tag.

**Supplementary Figure 10. Functional quantification and SOX4 re-expression assays.**

(A, B) Quantification of colony formation, EdU incorporation, wound closure, and Transwell cell counts in BCPAP and TPC-1 cells following OGT perturbation (A; Supplementary Figure 9F) or SERPINA1 overexpression and/or SOX4 knockdown (B; Figure 6K).

(C, D) Western blots and GAPDH-normalized quantification of FN1, SERPINA1, and SOX4 after SERPINA1 knockdown and/or SOX4 overexpression, matching the conditions in Figure 6J. Protein values were not further normalized to the control-group mean.

(E) LEC tube formation, Transwell migration, and wound healing under Control, CM, and Co-culture conditions (upper row; Figure 7C), or following tumor-cell SERPINA1 knockdown and/or FN1 overexpression (lower row; Figure 7F). Tube formation and wound healing are relative to control; Transwell values are cell counts. Wound closure was normalized to the matched control in each experiment.

Data are mean ± SD with individual values. Panels A and B include three observations per group; panels D and E represent three independent experiments per group. Four-group comparisons were knockdown versus control, combination versus knockdown, combination versus overexpression, and overexpression versus control. Panels A, B, and D used two-sided unpaired Welch t-tests with unadjusted P values for these four comparisons within each cell line and assay or protein. In E, tube-formation and Transwell data used two-sided unpaired Welch t-tests; control-normalized wound-healing data used two-sided one-sample t-tests against 1 for control comparisons and two-sided paired t-tests between treatments, matching corresponding replicate positions across groups. Holm correction was applied separately within each assay to all three upper-row pairwise comparisons or the four lower-row comparisons. ns, P ≥ 0.05; *P < 0.05; **P < 0.01; ***P < 0.001; ****P < 0.0001.

LEC, lymphatic endothelial cell; CM, conditioned medium; EdU, 5-ethynyl-2′-deoxyuridine; OGT, O-linked N-acetylglucosamine transferase; GAPDH, glyceraldehyde-3-phosphate dehydrogenase; SD, standard deviation; a.u., arbitrary units.
